# Correction: Detection through the use of RT-MqPCR of asymptomatic reservoirs of malaria in samples of patients from the indigenous Comarca of Guna Yala, Panama: Essential method to achieve the elimination of malaria

**DOI:** 10.1371/journal.pone.0329753

**Published:** 2025-08-04

**Authors:** Lorenzo Cáceres Carrera, Ana María Santamaría, Anakena Margarita Castillo, Luis Romero, Eduardo Urriola, Rolando Torres-Cosme, José Eduardo Calzada

In the Conclusion section, there is an error in the fourth sentence. The correct sentence is: This study highlights the need for longitudinal studies conducted in all endemic regions of Panama to evaluate the prevalence of asymptomatic infections.
