# Supplementary material for: Detection through the use of RT-MqPCR of asymptomatic reservoirs of malaria in samples of patients from the indigenous Comarca of Guna Yala, Panama: Essential method to achieve the elimination of malaria
Source: PLoS One. 2024 Jul 24;19(7):e0305558. doi: 10.1371/journal.pone.0305558 (PMC11268588; doi:10.1371/journal.pone.0305558)
Supplement: Attachment — Submitted filename: PONE-D-23-20941_reviewer_GMPA.pdf [file pone.0305558.s001.pdf]

PLOS ONE

Detection through the use of RT-MqPCR of asymptomatic reservoirs of malaria in samples of patients from the indigenous Comarca of Guna Yala, Panama: Essential method to achieve the elimination of malaria.

--Manuscript Draft--

**Manuscript Number:**	PONE-D-23-20941
**Article Type:**	Research Article
**Full Title:**	Detection through the use of RT-MqPCR of asymptomatic reservoirs of malaria in samples of patients from the indigenous Comarca of Guna Yala, Panama: Essential method to achieve the elimination of malaria.
**Short Title:**	Detection by RT-MqPCR of asymptomatic infection by P. vivax in Panama
**Corresponding Author:**	Lorenzo Cáceres Carrera, Ph.D<br>Instituto Conmemorativo Gorgas de Estudios de la Salud<br>Panamá, Panamá PANAMA
**Keywords:**	Malaria, Plasmodium vivax, asymptomatic, diagnosis, microscopy, PCR, prevalence, submicroscopic, sensitivity, Panama.
**Abstract:**	Background<br>Plasmodium vivax is the dominant species causing malaria in Panama. However, the prevalence of asymptomatic infections in the different endemic regions is unknown. Understanding the epidemiological behavior of asymptomatic infections is essential to achieve the elimination of malaria. The objective of the study was to determine the prevalence of asymptomatic malaria infections through RT-MqPCR in one of the main endemic regions of the country.<br>Methods<br>A cross-sectional study was carried out in three communities of the Guna Yala Comarca. For this, 551 thick blood smears and their respective sample on filter paper were collected from volunteers of different ages and sex during June 20-25, 2016. Infections by Plasmodium spp. they were diagnosed by microscopy and RT-MqPCR.<br>Results<br>The average prevalence of asymptomatic infections by P. vivax in the three communities detected by RT-MqPCR was 9.3%, Ukupa registered the highest prevalence with 13.4%, followed by Aidirgandi with 11.1% and Irgandi with 3.3%. A total of 74 samples were diagnosed with asymptomatic infections by RT-MqPCR. Light microscopy detected 17.6% (13/74) of the asymptomatic samples and 82.4% (61/74) were diagnosed as false negatives. A 100% concordance was recorded between the samples diagnosed with LM and RT-MqPCR. 52.7% (39/74) of the asymptomatic patients were female and 85.1% (63/74) were registered between the ages ˂1 - 21 years of age.<br>Conclusions<br>The RT-MqPCR presented a high sensitivity in the detection of asymptomatic infections by P. vivax in comparison with the LM, which proved to be an insufficient technique for the active detection of patients with low parasitemia. This study provides evidence for the first time of the existence of a significant prevalence of asymptomatic infections by P. vivax in one of the main endemic regions of Panama. This represents a new paradigm for the health authorities that requires immediate attention, with the purpose of maintaining the achievements obtained to date and being able to reach the new goal of eliminating malaria in the country.
**Order of Authors:**	Lorenzo Cáceres Carrera, PhD
	Ana María Samtamaría, MSc
	Anakena M Castillo, PhD
	Luis Romero, Bachelor
	Eduardo Urriola, Bachelor
	Rolando Torres-Cosme, Bachelor

Powered by Editorial Manager® and ProduXion Manager® from Aries Systems Corporation

	José E. Calzada, PhD
**Additional Information:**	
**Question**	**Response**
**Financial Disclosure**<br><br>Enter a financial disclosure statement that describes the sources of funding for the work included in this submission. Review the submission guidelines for detailed requirements. View published research articles from *PLOS ONE* for specific examples.<br><br>This statement is required for submission and **will appear in the published article** if the submission is accepted. Please make sure it is accurate.<br><br>**Unfunded studies**<br>Enter: *The author(s) received no specific funding for this work.*<br><br>**Funded studies**<br>Enter a statement with the following details:<br>• Initials of the authors who received each award<br>• Grant numbers awarded to each author<br>• The full name of each funder<br>• URL of each funder website<br>• Did the sponsors or funders play any role in the study design, data collection and analysis, decision to publish, or preparation of the manuscript?<br>• **NO** - Include this sentence at the end of your statement: *The funders had no role in study design, data collection and analysis, decision to publish, or preparation of the manuscript.*<br>• **YES** - Specify the role(s) played.<br><br>* typeset	LCC Received the financing<br>Ministerio de Economía y Finanzas de Panama<br>www.mef.gob.pa<br>The funders had no role in study design, data collection and analysis, decision to publish, or preparation of the manuscript.
**Competing Interests**<br><br>Use the instructions below to enter a competing interest statement for this submission. On behalf of all authors, disclose any competing interests that could be perceived to bias this work—acknowledging all financial support and any other relevant financial or non-financial competing interests.	The authors have declared that no competing interests exist.

This statement is required for submission and **will appear in the published article** if the submission is accepted. Please make sure it is accurate and that any funding sources listed in your Funding Information later in the submission form are also declared in your Financial Disclosure statement.<br><br>View published research articles from *PLOS ONE* for specific examples.<br><br>**NO authors have competing interests**<br><br>Enter: *The authors have declared that no competing interests exist.*<br><br>**Authors with competing interests**<br><br>Enter competing interest details beginning with this statement:<br><br>*I have read the journal's policy and the authors of this manuscript have the following competing interests: [insert competing interests here]*<br><br>* typeset	
**Ethics Statement**<br><br>Enter an ethics statement for this submission. This statement is required if the study involved:<br><br>• Human participants<br>• Human specimens or tissue<br>• Vertebrate animals or cephalopods<br>• Vertebrate embryos or tissues<br>• Field research<br><br>Write "N/A" if the submission does not require an ethics statement.<br><br>General guidance is provided below. Consult the submission guidelines for detailed instructions. **Make sure that all**	Institutional Technical Committee del Programa Nacional de Malaria del Ministerio de Salud de Panamá<br>Note No. 147/DCV/DGSP/MINSA<br>Indicate the form of consent obtained: oral

**information entered here is included in the Methods section of the manuscript.**<br><br>**Format for specific study types**<br><br>**Human Subject Research (involving human participants and/or tissue)**<br>• Give the name of the institutional review board or ethics committee that approved the study<br>• Include the approval number and/or a statement indicating approval of this research<br>• Indicate the form of consent obtained (written/oral) or the reason that consent was not obtained (e.g. the data were analyzed anonymously)<br><br>**Animal Research (involving vertebrate animals, embryos or tissues)**<br>• Provide the name of the Institutional Animal Care and Use Committee (IACUC) or other relevant ethics board that reviewed the study protocol, and indicate whether they approved this research or granted a formal waiver of ethical approval<br>• Include an approval number if one was obtained<br>• If the study involved *non-human primates*, add *additional details* about animal welfare and steps taken to ameliorate suffering<br>• If anesthesia, euthanasia, or any kind of animal sacrifice is part of the study, include briefly which substances and/or methods were applied<br><br>**Field Research**<br><br>Include the following details if this study involves the collection of plant, animal, or other materials from a natural setting:<br>• Field permit number<br>• Name of the institution or relevant body that granted permission	
**Data Availability**<br><br>Authors are required to make all data underlying the findings described fully available, without restriction, and from the time of publication. PLOS allows rare exceptions to address legal and ethical	Yes - all data are fully available without restriction

concerns. See the PLOS Data Policy and FAQ for detailed information.<br><br>A Data Availability Statement describing where the data can be found is required at submission. Your answers to this question constitute the Data Availability Statement and **will be published in the article**, if accepted.<br><br>**Important:** Stating 'data available on request from the author' is not sufficient. If your data are only available upon request, select 'No' for the first question and explain your exceptional situation in the text box.<br><br>Do the authors confirm that all data underlying the findings described in their manuscript are fully available without restriction?	
**Describe where the data may be found in full sentences. If you are copying our sample text, replace any instances of XXX with the appropriate details.**<br><br>• If the data are **held or will be held in a public repository**, include URLs, accession numbers or DOIs. If this information will only be available after acceptance, indicate this by ticking the box below. For example: *All XXX files are available from the XXX database (accession number(s) XXX, XXX.*).<br>• If the data are all contained **within the manuscript and/or Supporting Information files**, enter the following: *All relevant data are within the manuscript and its Supporting Information files.*<br>• If neither of these applies but you are able to provide **details of access elsewhere**, with or without limitations, please do so. For example:<br><br>*Data cannot be shared publicly because of [XXX]. Data are available from the XXX Institutional Data Access / Ethics Committee (contact via XXX) for researchers who meet the criteria for access to confidential data.*	All relevant data are within the manuscript and its Supporting Information files.

Powered by Editorial Manager® and ProduXion Manager® from Aries Systems Corporation

The data underlying the results presented in the study are available from (include the name of the third party and contact information or URL).<br>• This text is appropriate if the data are owned by a third party and authors do not have permission to share the data.<br><br>* typeset	
Additional data availability information:	Tick here if the URLs/accession numbers/DOIs will be available only after acceptance of the manuscript for publication so that we can ensure their inclusion before publication.

Powered by Editorial Manager® and ProduXion Manager® from Aries Systems Corporation

Manuscript
Click here to access/download;Manuscript;Asymptomatic malaria - July 2023.doc

Detection through the use of RT-MqPCR of asymptomatic reservoirs of malaria in samples of patients from the indigenous Comarca of Guna Yala, Panama: Essential method to achieve the elimination of malaria

Detection by RT-MqPCR of asymptomatic infection by *P. vivax* in Panama.

Lorenzo Cáceres Carrera[1]*, Ana María Santamaría[2], Anakena M. Castillo[1], Luis Romero[3], Eduardo Urriola[4], Rolando Torres-Cosme[1], José E. Calzada[2].

[1]Departmento de Entomología Médica del Instituto Conmemorativo Gorgas de Estudios de la Salud. Ciudad de Panamá, Panamá.

[2]Departmento de Parasitología del Instituto Conmemorativo Gorgas de Estudios de la Salud. Ciudad de Panamá, Panamá.

[3]Laboratorio Central de Referencia en Salud Pública del Instituto Conmemorativo Gorgas de Estudios de la Salud. Ciudad de Panamá, Panamá.

[4]Universidad Latina de Panamá. Facultad de Ciencias Biomédicas. Ciudad de Panamá, Panamá.

Corresponding author: Lorenzo Cáceres Carrera. Departamento de Entomología Médica del Instituto Conmemorativo Gorgas de Estudios de la Salud. Ciudad de Panamá, Panamá. Phone: [507] 527-4963. Email: lcaceres@gorgas.gob.pa and caceres lorenzo@gmail.com

Abstract

Background

Plasmodium vivax is the dominant species causing malaria in Panama. However, the prevalence of asymptomatic infections in the different endemic regions is unknown. Understanding the epidemiological behavior of asymptomatic infections is essential to achieve the elimination of malaria. The objective of the study was to determine the prevalence of asymptomatic malaria infections through RT-MqPCR in one of the main endemic regions of the country.

Methods

A cross-sectional study was carried out in three communities of the Guna Yala Comarca. For this, 551 thick blood smears and their respective sample on filter paper were collected from volunteers of different ages and sex during June 20-25, 2016. Infections by *Plasmodium spp*. they were diagnosed by microscopy and RT-MqPCR.

Results

The average prevalence of asymptomatic infections by *P. vivax* in the three communities detected by RT-MqPCR was 9.3%, Ukupa registered the highest prevalence with 13.4%, followed by Aidirgandi with 11.1% and Irgandi with 3.3%. A total of 74 samples were diagnosed with asymptomatic infections by RT-MqPCR. Light microscopy detected 17.6% (13/74) of the asymptomatic samples and 82.4% (61/74) were diagnosed as false negatives. A 100% concordance was recorded between the samples diagnosed with LM and RT-MqPCR. 52.7% (39/74) of the asymptomatic patients were female and 85.1% (63/74) were registered between the ages <1 - 21 years of age.

Conclusions

The RT-MqPCR presented a high sensitivity in the detection of asymptomatic infections by *P. vivax* in comparison with the LM, which proved to be an insufficient technique for the active detection of patients with low parasitemia. This study provides evidence for the first time of the existence of a significant prevalence of asymptomatic infections by *P. vivax* in one of the main endemic regions of Panama. This represents a new paradigm for the health authorities that requires immediate attention, with the purpose of maintaining the achievements obtained to date and being able to reach the new goal of eliminating malaria in the country.

Keywords: Malaria, *Plasmodium vivax*, asymptomatic, diagnosis, microscopy, PCR, prevalence, submicroscopic, sensitivity, Panama.

Introduction

Malaria is the most widespread and serious parasitic disease in the world [1]. The rapid reduction of malaria from the map brings us one step closer worldwide to eliminating this disease, yet great challenges remain. To achieve elimination and prevent its resurgence, surveillance systems need to adapt to the changing epidemiology of malaria and be able to detect all potential malaria infections in a timely manner. Therefore, the accurate identification of all types of malaria infections, including symptomatic and asymptomatic, has become a vital component of control and elimination programs [2]. Asymptomatic malarial infection refers to parasitemia of any density in the absence of fever or other acute symptoms in individuals who have not received recent antimalarial treatments [3]. Some asymptomatic infections have levels of parasitemia that are detectable by microscopy, while others can only be detected by molecular methods, and are called submicroscopic infections. At any given time, a large majority of individuals with detectable malaria parasitemia can be categorized as asymptomatic [4], and are considered important reservoirs sustaining malaria transmission [5]. However, there are situations in which the presence of asexual forms of the parasite is not associated with clinical malaria, for example, when the level of parasitemia is below the threshold at which an individual develops symptoms (pyrogenic threshold) or when the immune system keeps parasitemia levels low for long periods and, thus, the individual free of symptoms [6]. Furthermore, asymptomatic malaria can be observed as a result of intermittent administration of subtherapeutic levels of antimalarials [7]. Therefore, the use of highly sensitive and low-cost tools for the specific detection of the parasite would be very useful in the malaria elimination phase [8]. Clearly for a removal strategy, the

use of highly sensitive detection methods is necessary and of significant importance [9-10].

Strategies aimed at eliminating malaria will require increased surveillance and the use of highly sensitive tests capable of detecting asymptomatic and low-density infection. These infections are often well below 200 parasites/ml and are an important reservoir capable of transmitting malaria that must be detected and eliminated [11-12]. Light microscopy (LM) of thick blood films and Giemsa-stained smears of peripheral blood continue to be considered the gold standard test in the diagnosis of malaria [13]. These microscopic tests, along with rapid diagnostic tests (RDTs) based on the detection of parasitic antigens, are the most commonly used tests in malaria prevalence surveys. Ultrasensitive RDTs improve the detection sensitivity of patients with a parasitemia between 200 parasites/ml and 10,000 parasites/ml [14], but are still limited by their low input volume.

Different molecular tests are currently used for the detection of *Plasmodium* species. The sensitivity of PCR is due to its ability to detect unique and specific nucleic acid molecules and the use of concentrated DNA from a large sample. The widespread use of PCR-based assays, such as qPCR and multiplex real-time reverse transcription PCR (RT-MqPCR), have revealed a new picture of malaria prevalence, particularly in low-transmission areas [15-16]. Multiplex real-time reverse transcription PCR has improved the capacity for the detection of mixed *Plasmodium* infections and the detection of *Plasmodium* species in cases of low parasitemia, with a detection limit of less than 2

parasites/ml and has the advantage of simultaneous detection of multiple targets in a single run to increase the sensitivity and specificity of the test, compared to LM, RDTs, and conventional nPCR [17-18]. In a previous study where RT-MqPCR and nPCR were compared to detect *P. vivax,* it was shown that RT-MqPCR had higher sensitive than nPCR [19-20].

In Panama, the number of malaria cases has decreased in recent decades, as in many countries in the region. Reaching an annual average of 647 malaria cases between 2008 and 2018. However, during the last four years malaria has increased significantly, registering an annual average of 3,818 malaria cases between 2019-2022. Reaching a total of cases of malaria in 2022 of 7,112 cases of malaria [21], going back in malaria behavior 65 years ago at the beginning of the campaign of eradication of malaria in 1957. Until now, there is no diagnosis or technical report that indicates which have been the main factors that have incurred in the increase in the incidence of malaria in a significant way and which may be the strategies to be used to confront the epidemic and reduce cases. To be able to confront the incidence of malaria, it is necessary to know the dynamics of transmission in endemic sites, mainly in foci with constant transmission and its potential to cause epidemic outbreaks in the different endemic and non-endemic regions in Panama. In turn, this will depend on being able to determine, among other important factors, the species of mosquitoes incriminated in the transmission of malaria, the state of resistance to insecticides in vector mosquitoes, the behavior of infections at the local level, the presence of asymptomatic malaria and the effectiveness of antimalarial strategies. Therefore, studies are needed to generate scientific evidence

that can be translated into efficient and effective interventions for its control and thus achieve the elimination of malaria. Currently, it is of great importance to investigate the clinical and public health importance of asymptomatic patients as a source of infection for the maintenance of malaria. We hypothesize that asymptomatic infections represent an important source of infectious gametocytes for vector mosquitoes that maintain malaria transmission in endemic regions.

The objective of this study was to determine, through the use of RT-MqPCR, the prevalence of asymptomatic malaria infections in the indigenous population of three communities belonging to the Comarca (The term comarca refers to an administrative region within Panama, assigned as a reserve to a certain indigenous population) of Guna Yala (CGY) that may be contributing as reservoirs of the parasite and maintaining the transmission of the disease. The scientific evidence found will contribute to improving and strengthening the policies for the elimination of the disease by the National Malaria Program (NMP) of the Ministry of Health (MINSA).

Method

Selection and description of study sites

The selection of the communities of Irgandi 9020'05.66" N; 78018'19.49" W; 7 meters above sea level (masl); 300 inhabitants, Ukupa 9021'21.80" N; 78019'56.21" W; 3 masl; 320 inhabitants and Aidirgandi 9023'05.56" N; 78025'12.56" W; 8 masl; 190 inhabitants (Fig 1), was carried out jointly with the NMP of MINSA and based on the information

provided on the magnitude of the malaria problem in Panama and particularly in the endemic regions of the country. The three selected communities are located within the CGY in the northeastern part of the country and on the border with Colombia (Fig 2-A).

Historically, the CGY has been an endemic region for malaria (Fig. 3), and as in the rest of the country, the species of parasitic infection that prevails is *P. vivax.* In the three localities, the residents are 100% indigenous of the Guna ethnic group and have access to malaria diagnosis and treatment through the technical staff of the NMP and the health facilities. The CGY registers an approximate population of 31,557 inhabitants and comprises approximately 2,340.7 km^2 of territorial surface, including approximately 480 km of coastline surrounded by reefs and mangroves, and around 365 small coral islands, whose ecology has been strongly modified by anthropogenic influence [22]. It presents a tropical forest ecology, with lowland areas in the coastal zone that are used by the Guna indigenous population to cultivate mainly coconuts and other crops. The average monthly temperature in the region is 26 to 27 ^{0}C, relative humidity between 78 and 90%, and annual rainfall that ranges between 1,600 and 3,000 mm [23]. The CGY region normally has a unimodal rainfall pattern with a dry effect. The dry season starts from mid-December to April, and a rainy season from May to mid-December.

Study Design

A cross-sectional study was conducted in three selected endemic communities with collaboration and participation of technical personnel from the NMP of MINSA (Fig 4) to determine the population prevalence with asymptomatic *P. vivax* and *P. falciparum*

malaria. The detection of the species *P. ovale* and *P. malariae* was not considered in this study since they have not been reported for 41 years in Panama [24]. Thick blood samples and filter paper were taken by finger puncture from all persons of different ages and sex (children and adults) who did not present any type of clinical signs or symptoms of malaria and without a history of fever in the last 72 hours at the time of sampling. Only people who had not left the town for at least the last 15 days were considered. The collection of samples was carried out in the period of time that coincided with the registration of the transmission activity of the disease with the beginning and the peak of the malaria season in this region of the country. All blood samples were taken by the vector control technical personal of the MINSA of the NMP.

Population under study

The study included a sample size that represented a significant percentage of the population living in each of the three selected communities. A representative sample between 40 and 50% of the population of each of the selected communities was established, which allowed a greater degree of reliability of the results to demonstrate the patterns of asymptomatic people with respect to the rest of the universe. The samples included all people of different ages and sex who agreed to participate voluntarily and who lived in the selected communities. The participation of minors had the consent of their parents or tutors. Asymptomatic subjects were those without fever (temperature <37.2 °C) and without a history of fever in the last 72 hours. Demographic data such as age, sex, place of residence, occupation, length of residence in malaria-endemic regions and history of disease, previous episodes of malaria and antimalarial

drugs used in the last month were obtained through the NMP of MINSA. The epidemiological information was obtained from the databases provided by the NMP and the Department of Epidemiology (DE) of MINSA.

Operational definition of asymptomatic infections by *Plasmodium spp*.

People who presented malaise, fever, chills, muscle pain and headache, in addition to positive LM and PCR were considered symptomatic. For the purposes of this study, asymptomatic infection by *Plasmodium spp*. were such as the detection by LM of parasitic asexual stages of *P. vivax* and/or *P. falciparum* or mixed infections in blood, in individuals who have not received, recent anti-malarial treatment, which persisted during blood sampling without presenting any symptoms, or as the detection of parasite DNA by RT-MqPCR real time in people who remained asymptomatic during the blood sample collection. Individuals with negative LM, but with subsequent evidence of positive PCR were later visited to provide antimalarial treatment.

Exclusion criteria

All asymptomatic people were considered to participate voluntarily in the study, with the exception of those who presented the following: 1) patients who had received antimalarial therapy in the last four weeks; 2) individuals with positive LM who remained without symptoms for 15 days, 3) people with symptoms of fever during the last 48

hours, 4) participants who reported being sick at the time of sampling, and 5) people who expressed not wanting to participate in the study.

Blood sample collection

The study included adults and minors. The collection of thick blood films, filter paper and data collection from the participants was carried out by highly experienced technical personnel from the NMP of MINSA from June 20 to 25, 2016. Sampling blood tests in the three communities were carried out with all family members who agreed to participate and did not show signs and symptoms of malaria. Before taking the blood sample, a brief routine questionnaire was carried out to find out the travel history and the existence or not of previous episodes of malaria in the last year, while basic information on name, age and sex was documented technical personnel from the NMP of MINSA as a routine record when taking a blood sample from a person suspected of malaria. The thick blood film and filter paper samples were obtained through a digital puncture with a sterile lancet on the participant's index finger (Fig 2-B and C). The blood samples were coded, recorded on a form, and carefully stored in sealed bags for later analysis at the ICGES Department of Parasitology.

Microscopic diagnosis

All thick blood samples were analyzed by two expert microscopists who are part of MINSA's NMP, each with more than 35 years of experience. Its ability to accurately diagnose blood samples is validated annually by Pan American Health Organization (PAHO) External Performance Evaluation Program for Malaria Microscopic Diagnosis. In

addition, it is the personnel that carries out the quality control of the diagnostic samples at the national level carried out by the Central Reference Laboratory for Public Health (LCRSP) of the ICGES. Malaria is a notifiable disease in Panama [25]. Each thick film sample and blood smear or peripheral smear was stained with Giemsa [26]. All samples were observed with LM, with 1000X magnification. The morphological characteristics of the different stages of *Plasmodium* maturation were differential criteria for the diagnosis of *P. vivax* and *P. falciparum* by LM. The final diagnosis was made after observing 100 fields before declaring a sample negative or positive for *Plasmodium spp.* [27-28]. Positive samples after reading 100 fields were transformed into parasites/µl of blood [29].

Diagnosis by RT-MqPCR

All DNA samples were analyzed with a commercial RT-MqPCR test for the detection and differentiation of human Plasmodium species (FTD Malaria, Fast-Track Diagnostics®, Esch-sur-Alzette, Luxembourg). For the amplifications, 10 µl of the extracted DNA were used and the manufacturer's instructions were followed (FTD Malaria differentiation Manual). The presence of specific sequences was detected by an increase in fluorescence observed from the relevant double-labeled probe, and reported as a cycle threshold value (Ct) using the ABI7500 Real-Time Thermal Cycler (thermo Fisher Scientific).

Treatment of malaria cases

The supply of antimalarial drugs is rigorously controlled by MINSA. The treatment is provided and strictly supervised by the technical staff of the NMP. All patients who were diagnosed with *P. vivax* infection by microscopy and/or positive PCR received full treatment based on 1,500 mg of chloroquine and 210 mg of primaquine for seven days supplied by NMP technical personnel. According to the NMP norm, at 15 days and six months a new blood sample is taken and examined to confirm that the case was cured [25].

Ethical considerations

This study was carried out at the request of the NMP of MINSA and was approved by the Institutional Technical Committee of the NMP of MINSA (Note No. 147/DCV/DGSP/MINSA). This study took into consideration the laws and traditions of the Guna indigenous culture. For this, meetings were previously held with the traditional indigenous authorities (Sahila) of each of the selected communities to explain the objectives and potential benefits of the study in order to obtain the approval of the study by the local indigenous authorities (Fig 2-D). The study began after each participant received detailed information from the MINSA technical staff on the objectives of the study and the verbal consent of each participant was obtained. In the case of minors, the consent of the parents or legal guardians was obtained.

Statistical analysis

For the study, a sample size of 40% of the population living in each of the three selected communities was considered, a percentage that was calculated with the Epi Info TM version 7.2 program. For the effect of taking a blood sample, a sample of between 40 and 50% was taken into account, which allowed evidencing patterns with respect to the rest of the universe, but, since the universe was relatively small, it was preferred to do the survey in 50% to have a higher degree of reliability in the results. In the sampling scheme, an attempt was made to the largest possible number of participants and thus the entire population had the same probability of being included in the sample; the confidence level was established at 99% and the margin of error at ± 0.03 %.

The population was characterized according to basic demographic variables such as age, sex, place of residence, and time spent in the community. The other variables of interest referred to a history of malaria infections during the last year, the treatment received, the species of parasite during the last episode, and the results of microscopic diagnosis and PCR. The database was saved in MS Excel ™ 2003; data were analyzed using Epi Info TM version 7.2 program. and STATA™ 7.0 was used to determine confidence intervals. To determine significant differences in the asymptomatic prevalence between communities and groups of individuals (age and sex), we use a Chi-square test (X^2).

Results

Analysis of the study population

Between June 20 and 25, 2016, a total of 551 thick blood samples for microscopic diagnosis and their corresponding sample on filter paper for the molecular diagnosis of *P. vivax* and *P. falciparum* of malaria asymptomatic patients were taken from people who voluntarily participated in this study. The mean age of the participants was 20.4 years and the median was 13 years, with a standard deviation of 17.5. The age range was <1 to 88 years of age, 64.2% (354/551) of the people sampled were between the ages <1 - 20 years, within this age group 37.4% (206/551) correspond to the age group <1 - 10 years, 28.5% (157/ 551) to the group 11 to 20 years and the rest of the samples taken in the other age groups. The samples taken in the three selected communities corresponded to the female sex with 54.6%.

In the Irgandi community, 179 blood samples were taken (179/551: 32.5%; 95% CI: 28.6 – 36.4), the mean age was 21.4 years, the median was 13 years, the standard deviation was 19.3, and the range age ranged from <1 to 88 years. 59.2% of the samples taken corresponded to the female sex. In Ukupa, a total of 211 blood samples were taken (211/551: 38.3%; 95% CI: 34.2 – 42.4), the mean age was 19.2 years, the median 12 years, a standard deviation of 16.3 and the age range was <1 - 69 years. 52.6% of the samples taken corresponded to the male sex. For its part, in Aidirgandi, a total of 161 blood samples were taken (161/551: 29.2%; 95% CI: 25.4 – 33.0), the mean age was 20.8 years, the median 13 years, one standard deviation 17 and an age range of <1 - 75

years. 59% of the samples taken corresponded to the female sex (Table 1). A large percentage of 74.4% (n = 410) of the blood samples obtained in the three selected communities corresponded to the population between the ages of <1 - 20 years of age. Table 1 also shows the distribution of the percentage of blood samples obtained in the population by locality, in Irgandi 59.7% of blood samples were obtained (179/300 inhabitants; 95% CI: 52.5 - 66.9), in Ukupa it was 66% (211/320 inhabitants; 95% CI: 60.8 – 71.2) and in Aidirgandi it was 84.7% (161/190 inhabitants; 95% CI: 79.1 – 90.3).

Diagnosis by microscopy and RT-MqPCR

All 551 thick blood smears and Giemsa-stained smears of asymptomatic people obtained in the sampling in the three selected communities were analyzed by LM; a total of 13 cases of people were asymptomatic for *P. vivax,* with a 2.4% of cases detected (13/551). Light microscopy diagnosed 17.6% (13/74) of asymptomatic cases detected by RT-MqPCR. No asymptomatic *P. falciparum* infections were detected in the blood samples analyzed. The parasitemia of the asymptomatic cases diagnosed by microscopy came to vary significantly according to the parasite density, being classified between low to moderate according to the level of parasitemia, with a range of 26 parasites/µl blood to 3,571 parasites/µl blood. Most of the asymptomatic individuals showed very low parasitemia and consequently low parasite counts (<500 parasites/µ[illegible] of blood). Within those diagnosed positive by microscopy, five blood samples presented gametocytes, with a range of 3 to 30 gametocytes (Table 2). Taking RT-MqPCR as the gold standard, LM detected only one sixth of asymptomatic infections. The highest percentage of asymptomatic infections detected by LM were in patients between the

ages of < 1 [illegible] 20 years of age (n = 11, 84.6%), the mean age was 11.8 years, the median 10 years, and the range < 1 to 37 years.

Microscopy versus RT-MqPCR

All samples (n = 551) were analyzed using RT-MqPCR. In the three communities studied, the percentage of detection of *P. vivax* infection by RT-MqPCR was 13.4% (74/551), much higher than by LM, which was 2.4% (13/551). The highest prevalence of asymptomatic infections by *P. vivax* detected by RT-MqPCR was in Ukupa with 13.4% (43/320) and while by LM it was 2.2% (7/320), followed by Aidirgandi with 11.1% (21/190) and LM was 3.2% (6/190). Finally, in Irgandi the lowest prevalence was detected by RT-MqPCR with 1.8% (10/300), by LM no asymptomatic cases were detected (Table 3). Asymptomatic *P. falciparum* infections could not be detected by RT-MqPCR and LM.

The community of Ukupa was the one that registered the largest number of asymptomatic patients due to *P. vivax* detected by RT-MqPCR, with 43 cases (43/79, 54.4%), where 51.2% (22/43) of the positive samples were female. In Aidirgandi, the female sex represented 52.4% (11/21) of the samples asymptomatic by *P. vivax,* respectively. Finally, in the community of Irgandi, the female sex represented 70% (7/10) of the positive samples of asymptomatic patients (Table 3). In Irgandi, no cases of people with asymptomatic *P. vivax* infections were diagnosed by LM. It was not possible to detect people with malaria symptoms at the time of sampling. None of the participants had left their communities during the last 20 days. With the use of RT-MqPCR there was

an increase in the proportion of positivity compared to LM, registering almost six times more positivity than the diagnosis by LM (1:5.7). Chi square showed significant difference in asymptomatic patients by sex ($X^2 = 3.85$, $p < 0.05$) and by communities, ($X^2 = 21.44$, $p < 0.001$), there was no significant difference by age.

Discussion

Incidence of malaria in the CGY Region

In CGY one of the most important variables that determines the intensity and prevalence of malaria in this region is the internal migration in the region of native people and the arrival of immigrants from South America who are symptomatic and asymptomatic carriers of the parasite where the communities are receptive and vulnerable to malaria cases or outbreaks. In this region, the malaria transmission dynamics is characterized by originating seasonally through small foci or epidemic outbreaks of transmission whose incidence is closely related to migratory movements, unprotected homes, anopheline breeding sites near homes, climate, conditions environmental, socioeconomic and cultural factors among others. The population has a low collective immunity, therefore, a high probability of epidemics [30]. Comarca Guna Yala is characterized by being historically endemic, where *P. vivax* infections and periodic appearances of *P. falciparum* predominate, mainly imported cases in recent years. This disease has always registered an endemic behavior, from 1965 to 2022 this region has contributed 15.1% of the total cases registered throughout the country [21].

The results of this study clearly demonstrate the existence of asymptomatic infections by *P. vivax* for the first time in Panama, in one of the main endemic regions in the country such as CGY. *Plasmodium vivax* was the only infecting species that prevailed in the asymptomatic cases detected in this study. *P. vivax* is the main parasite that causes malaria in the country. From 2000 to 2022 through the reports of malaria cases from the NMP, a total of 42,683 cases of malaria have been registered throughout the country, where 92.1% of reported malaria cases have been caused by *P. vivax* [21]. The prevalence of *P. vivax* in Panama is consistent with the distribution of malaria parasite species historically observed in this region. *Plasmodium vivax*, the predominant parasite species causing malaria in the Americas, are extremely diverse genetically and geographically, with significant genetic divergence between populations separated by relatively short distances [31-32].

This finding in CGY of a significant prevalence of asymptomatic people due to *P. vivax* may explain the maintenance or persistence of residual foci of transmission due to the existence of asymptomatic human reservoirs with parasitemias that are not regularly detected by LM. This situation reverses the history of outbreaks and the maintenance of epidemics in CGY, in the sense that native populations may be significantly incriminated with the maintenance of malaria transmission and not only the movements of symptomatic and asymptomatic immigrants are the responsible for the constant presence of malaria in this region. First, the prevalence of asymptomatic infections has been shown to be highly significant in all three CGY communities. This fact had not been recognized before the use of PCR because only the diagnosis was made by LM

and this method only detects a fraction of the positive cases for malaria. People with undetected asymptomatic infections do not seek care at health services or NMP staff and do not receive antimalarial treatment. Thus, only clinical cases of malaria are recorded, while a significant percentage of malaria infections of the native populations are without symptoms, thus contributing to the maintenance of the transmission cycle through the participation of anopheline vectors and therefore, the maintenance of the disease. In addition, our results demonstrate a greater effectiveness of PCR to detect these asymptomatic parasitemias compared to LM.

Asymptomatic patients with submicroscopic parasitemia may sustain continuous malaria transmission, even under low transmission conditions, if they are not detected by a highly sensitive method and promptly treated with effective antimalarial drugs [33-34]. The detection of asymptomatic infections [35] and the demonstration that asymptomatic people carrying parasites can infect vector anopheline mosquitoes [36], raises the role that those asymptomatic patients play in the dynamics of transmission and maintenance of the disease. Consequently, treatment of asymptomatic and submicroscopic infections can be of fundamental importance to improve antimalarial strategies and move towards their elimination [34]. In contrast, asymptomatic parasitemia is considered relatively uncommon in areas of low seasonal transmission that predominate in much of Asia and the Americas. Recent epidemiological studies more frequently use sensitive molecular methods such as PCR to estimate the true burden of malaria in areas endemic species to plan effective control methods against this disease [37-39]. The availability of an efficient and effective technique to detect asymptomatic infections may be a key factor

for decision-making by the NMP in the fight against malaria and its elimination. There needs to be a strategy to detect and reduce asymptomatic cases and submicroscopic infections in order to eliminate malaria in the endemic regions of the country. This observation is in agreement with reports from similar studies conducted in Sri Lanka [40-41], Iran [42], Solomon Islands [43] and Paraguay [44]. All these reports suggest the reduction of asymptomatic cases, submicroscopic parasitemia and the reduction of symptomatic cases of malaria in the population at risk to achieve the elimination of malaria.

Analysis of the population studied

In this study we observed that a high percentage of the blood samples taken in the three communities corresponded to ages <1 - 20 years of age (n = 363, 65.9%), within this age group, a significant percentage of the blood samples was taken between the ages of <1 - 10 years of age (n = 206, 37.4%). This group of <1 - 10 years of age, had a correspondence with the significant prevalence of asymptomatic patients with *P. vivax* infection diagnosed by RT-MqPCR in the three communities (38/810, 5.0%). Regardless of the age range with the highest prevalence, this study has revealed the silent presence of asymptomatic reservoirs of the malaria parasite *P. vivax* in different age groups in three communities, which may be contributing significantly to maintaining the transmission of the disease in CGY. Among demographic factors, female gender and children in the <1-10 age group were associated with asymptomatic *P. vivax* infections. This fact indicates that the greatest risk of contracting asymptomatic malaria was found within this age group and the probabilities decrease with increasing age. In a study

carried out in CGY, it was observed that 27.3% of the children positive for malaria were under 10 years of age, registering a significant percentage in terms of the incidence of the disease [30]. These results are consistent with similar observations made in Ethiopia and Peru [45-46]. It has been argued that this population group has a high probability of exposure to competent vectors and a higher risk of acquiring malaria infection. Previous work indicates that this asymptomatic age group poses many problems because they are unknown carriers of the parasite and that these reservoirs come to contribute to the maintenance of the life cycle of the parasite and the maintenance of disease transmission, as documented in communities endemic to malaria [47-48]. However, it should be noted that all age groups were exposed to asymptomatic *P. vivax* infections; the results of this study and other similar works confirm this situation. Among the results of this study, it was observed that the age group <1 - 10 years recorded 51.4% of the asymptomatic cases detected, this result may indicate that this age group is more likely to be asymptomatic to malaria infections. This result is similar to studies conducted in Africa, where school-age children were more likely to have acute *P. vivax* malaria [49-50]. Other recent similar studies revealed that school-age children showed higher levels of asymptomatic infection or gametocyte carriers. This higher prevalence of asymptomatic infections in underage children could be the result of increased exposure to *Plasmodium* due to inappropriate use of bed nets and antimalarial treatment [51-52].

The population studied is relatively stable and are residents for many years in the three communities studied. The three communities are located on the seashore and with rivers or streams close to them with a diversity of anopheline vector breeding sites. The

distribution of asymptomatic patients showed a slight concentration in the central part of the studied communities, the rest of the asymptomatic patients were dispersed within each one of the communities. The entomological variables that could explain this distribution pattern remain to be studied. The percentage of asymptomatic infections by *P. vivax* detected by RT-MqPCR was higher in females in the community of Irgandi (n = 6; 60%), followed by Aidirgandi (n = 11; 52.4%) and in Ukupa it was of (n = 22; 51.2%). While children between <1 to 10 years of age represented (n = 38, 51.4%) of the asymptomatic cases. This can probably be due to the permanence of adult women and minor children in the homes, to the activities carried out in the intra and peri-domestic environment during the period of highest frequency of bites of anopheline vectors and the proximity of the breeding sites located in rivers and streams near these communities. Studies carried out indicate that the high rate of asymptomatic malaria infections in women and minor children may be due to exposure to mosquito bites in various types of environments, densities of adult mosquitoes, location of houses in relation to breeding sites of mosquitoes, low immunity, unprotected housing, lack of knowledge and negative health behaviors [53-55]. It is important to indicate, according to the results of the study, that the percentage of asymptomatic malaria found showed an almost similar spatial behavior in the three communities, possibly due to the existing proximity between the three communities. This may also indicate why the epidemiological behavior of malaria is almost the same, since historically these three communities have always registered cases of malaria in CGY. Based on the results obtained in this study, it can be said that the probability of being asymptomatic due to malaria was higher in minor children than in adults (n = 19, 25.7%), suggesting that age

could be an important risk factor for the presence of asymptomatic infections. Similarly, other studies have reported that Children can act as a reservoir for parasites and facilitate malaria transmission. This can have serious implications in underage children, as it can affect brain development, academic performance [56-57] and parasitemia can lead to severe malaria [58-59]. It can be indicated according to the results that malaria control intervention initiatives should be directed considerably and specifically at underage children in order to achieve the elimination of malaria throughout the entire CGY Shire.

Microscopic diagnosis

The accurate diagnosis of *Plasmodium* species is important not only to establish the correct antimalarial treatment, but also to apply effective malaria control strategies in endemic regions where there are more than one species of malaria parasites, such as CGY. Malaria control requires a high-quality diagnostic method to detect the parasite species before prescribing antimalarial treatment, considering the indications of the WHO. The parasitological diagnosis of malaria points to the selection of an adequate treatment, supports the characterization of the response to treatment and allows early identification of the parasite and timely treatment [60].

In Panama, malaria is mainly caused by *P. vivax,* which accounts for more than 90% of diagnosed cases of the disease [61]. In this study, LM diagnosis detected only 17.6% of asymptomatic *P. vivax* malaria diagnosed by RT-MqPCR in the three communities. Through previous studies, it has been known that LM has missed approximately 50% of

all PCR-positive malaria infections [62]. The microscopic diagnosis varies considerably between <10 - 500 parasites/µl of blood, microscopy is reliable if there is a high density of parasites. In the presence of low parasite densities, a negative result does not exclude the presence of malaria infection [63,9]. The effectiveness of LM for malaria diagnosis depends on maintaining a high level of staff competence and accurate performance at all levels [64].

In this study, among the individuals with positive asymptomatic infection by microscopy, five patients showed the presence of gametocytes in low numbers (3-30 gametocytes), gametocytes are important for the maintenance of malaria transmission. In studies carried out on individuals positive with *P. vivax* gametocytes, they showed that the proportion of asymptomatic individuals was lower. This may be explained by the lower probability of detecting gametocytes in low densities of blood stage parasites, as seen in asymptomatic individuals. Although cases with lower parasitemia and, therefore, lower gametocythemia have been detected, it has been shown that asymptomatic infections are capable of infecting mosquitoes [36] and, therefore, could be an important reservoir, especially for seasonal outbreaks [65]. A study in the south of the Amazon state estimated that 54.4% of all parasite biomass belonged to infected asymptomatic individuals, which accounted for 56.6% of all infections [66]. Other reports show a higher rate of gametocyte carriers; however, these were symptomatic patients who had high parasite densities that correlated with higher gametocyte detection [67-69].

Malaria is a public health problem in CGY, *P. vivax* parasitic infections account for more than 90% of malaria cases historically and it is a notifiable disease [25,30]. In the present study, LM showed less sensitivity than RT-MqPCR, which was highly effective in diagnosing asymptomatic patients due to *P. vivax.* Considering the age groups, a decrease in sensitivity was observed with age in the microscopic diagnosis. About the density of the parasite registered, it is determined by the positive infections detected by LM that was between low and moderate (26 to 3,571 parasites/µl of blood), in works carried out it has been indicated that the density of the parasite decreases with age [70].

This study highlights the low effectiveness and possible deficiencies of the diagnosis of asymptomatic malaria patients by LM in the three communities, if PCR is considered as the gold standard to compare the results obtained by LM, a high percentage of false negatives of 82.4%. was found by LM. False negative LM results are known to increase as the parasite density decreases [71]. False negatives are a major public health problem because there is a part of the population that returns home without correct diagnosis and treatment, it is not possible to carry out a rapid, correct diagnosis and treatment with the confirmed presence of parasites. This could have important implications for health, transmission, and possibly the mortality record. Accurate diagnostic methods are the basis for proper disease control and avoiding resistance to antimalarial drugs or the spread of resistance and disease. The low density of parasites, together with the high number of false negatives detected by microscopy, indicates that it is often difficult to give a good microscopic diagnosis [72]. Although LM has been recommended by the WHO as a gold standard in the diagnosis of symptomatic patients,

many studies have reported that at low parasite densities, especially among asymptomatic carriers, parasites are easily missed by this method due to Inherent limitations such as lack of experience in microscopic diagnosis, sample preparation method, staining technique, and detection limits [73-76]. Despite these limitations, microscopic diagnosis through thick blood film and peripheral blood smears remains the "gold standard" for diagnosis of parasites for NMP today [77,25].

Diagnosis by RT-MqPCR

In this study, 551 samples from people who voluntarily participated in the three selected communities were analyzed by RT-MqPCR, with the result of 74 positive samples from asymptomatic individuals by this molecular technique. Many (n = 61, 82.4%) asymptomatic patients detected by RT-MqPCR were false negative by LM, showing that RT-MqPCR was more effective in detecting asymptomatic patients or submicroscopic parasitemia by *P. vivax,* showing a high sensitivity and specificity. It is important to indicate that in Panama, the use of PCR is not applied as a diagnostic method by public health services. Therefore, the routine or sporadic use of this ultrasensitive molecular technique by public health services can contribute significantly to the detection of submicroscopic and asymptomatic infections that cannot be detected by LM used as a standard method by the NMP, taking into consideration that asymptomatic infections contribute substantially to the transmission and maintenance of malaria in endemic and non-endemic regions. In a previous study carried out on the characterization of a malaria epidemic outbreak registered in CGY, it was indicated that due to the possible participation of symptomatic and asymptomatic immigrants from South America and

natives of the guna population, they could be participating in the maintenance of transmission. of the disease in CGY, it was indicated that the use of sensitive molecular methods was necessary to detect patients with asymptomatic infections and thus determine the real burden of malaria in the three communities studied [30]. It is important to indicate that the detection of asymptomatic cases is not part of malaria programs. To address this problem, it is necessary for health policy makers to develop collaboration strategies with scientific centers and institutions to detect asymptomatic cases of malaria in order to achieve effective control and elimination. The results of this study highlight the need for the NMP to incorporate more sensitive malaria diagnostic methods, such as the use of PCR molecular tests to characterize endemic and non-endemic regions.

The results of this study have been consistent with previous studies that found that LM has lower sensitivity for detecting *Plasmodium spp*. compared to molecular diagnostic methods such as nPCR [78], multiplex nested PCR (NM-PCR) [72] and RT-MqPCR [79]. PCR is the most common molecular method used for the detection of asymptomatic malaria. In the Americas, reports of asymptomatic infection are relatively recent. In Brazil, it was shown that 70% of cases among mineral prospectors in Mato Grosso were asymptomatic [80]. Among indigenous populations in the Colombian part of the Amazon basin a percentage of 21.6% was found of asymptomatic [81-82]. In the Peruvian part of the Amazon basin, a prevalence of 17.6% was found [83], and in the middle and upper reaches of the Río Negro it was shown that 20.4% of cases among the population involved in mining activities were asymptomatic [84]. On the other hand, a study carried

out in Venezuela detected a prevalence of infection of 1.3% by RDTs, 8% by LM and PCR allowed the detection of 10 asymptomatic cases, with a sensitivity and specificity of 100% and 93.4% respectively [85]. In Colombia, a prevalence of asymptomatic patients detected by qPCR of 10% in 2011, 7% in 2013, and 4% in 2014, respectively, was observed, while by LM the prevalence was <0.2%. The predominance was of *P. vivax* in the study [86]. In Peru using qPCR and RT-MqPCR, respectively, it was observed that 59.8% of all *P. vivax* gametocyte carriers were asymptomatic and 31.9% were submicroscopic [87].

Molecular detection methods, although not suitable for large-scale operational field work, are normally applied to obtain a representative assessment of the malaria situation in a subpopulation of a given area or region. With ease and speed of detection, PCR is commonly used, and the detection limit is generally 50-100 times that of LM and RDTs [88-91]. A CLIP-PCR has recently been developed with a sensitivity level as low as 0.01 parasites/µl of blood and a much higher throughput that could be suitable for active detection of malaria parasites in low transmission sites [92]. The PCR has shown in several previous studies in different countries that it presents a higher sensitivity than the LM and serological methods for the detection of *Plasmodium spp.* in symptomatic and asymptomatic patients, this characteristic being extremely important for the correct treatment of the patient with the advantages of timely epidemiological controls [93-95].

In order to achieve effective control and move towards the elimination of malaria in Panama, the precise epidemiological characterization of symptomatic and asymptomatic

malaria carriers and their geographical distribution is necessary. Their results are essential to be able to develop effective strategies against the transmission of the disease. disease in different endemic and non-endemic regions to achieve its elimination. Several studies have indicated that PCR diagnosis is considered the most ideal method to identify mixed infections [96], as well as *P. falciparum* and *P. vivax* infection [97-98]. The results of the detection of asymptomatic *P. vivax* infections by RT-MqPCR indicate that the sensitivity and specificity values in this study were optimal in accordance with those reported by other authors [99-100]. Once the results were obtained through the diagnosis by LM and RT-MqPCR of the cases of patients with asymptomatic malaria infection by *P. vivax,* the NMP was notified for the follow-up of each one of the patients. All asymptomatic patients were successfully treated, and each case was followed up according to the national standard for therapeutic management of patients with *P. vivax* malaria. Chemoprophylactic treatment is aimed at clinical cure, that is, the elimination of clinical signs and symptoms, the prevention of relapses, or radical cure, that is, clinical cure with the elimination of parasites [101-102].

Through the results of this study, the first report on asymptomatic malaria infections in Panama is made, demonstrating the silent presence of a population with asymptomatic infections by *P. vivax* in CGY, a region that has historically been endemic. This same epidemiological situation may be occurring in the different sites of active malaria transmission in the country. The significant prevalence found in the three communities of *P. vivax* asymptomatic patients is of fundamental importance because these asymptomatic patients contribute to the maintenance of disease transmission. This

situation brings therefore a new paradigm for the NMP that requires immediate attention, in order to maintain the achievements obtained in the fight against malaria to date and achieve the new goal of eliminating the disease in the country. Until the present study, asymptomatic infections had never been described in Panama, it was only mentioned a possible immunological state that people living in the different endemic regions develop [30,103]. It was also possible to observe that a considerable number of asymptomatic infections are not detected by LM used as a standard diagnostic test for malaria by the NMP. These results support the need to implement more sensitive diagnostic techniques to detect asymptomatic malaria. As observed, the molecular method of RT-MqPCR was sensitive enough to carry out an active search to determine the prevalence of asymptomatic *P. vivax* infections in three communities and a greater detection capacity than LM, making it very useful for epidemiological studies. This research work was not designed to determine the immunological mechanisms that may be behind asymptomatic malaria. Despite the nature of the underlying immune mechanisms that may be present in a population from an endemic region, several arguments have been raised on this subject, however, it was not raised in this study because this phenomenon was considered complicated to develop this type of research work and relate it to asymptomatic malaria.

There is a need to carry out new studies in CGY that include entomological aspects and evaluate the immune response for a better understanding of asymptomatic and symptomatic infections by *Plasmodium spp*. and its contribution to the dynamics of malaria transmission. Likewise, it is necessary to carry out studies in other endemic

regions of the country, with representative samples in order to establish the potential role of asymptomatic infections in the general prevalence of malaria, genotyping of asexual and sexual parasites, seasonality, variability and their contribution to the incidence of malaria. the disease in each endemic region. All the evidence generated must be provided to the NMP of MINSA so that they consider the threat that asymptomatic malaria represents for the national malaria elimination agenda and can be translated into effective interventions for control and in the immediate future to achieve the elimination of malaria. from the country.

Limitations

The main limitation of this study is that it had a limited geographical coverage and, therefore, generalization about the behavior of the prevalence of asymptomatic infections in the different endemic regions cannot be possible. Furthermore, the study was based on a cross-sectional design and therefore had limitations in establishing any type of causal association between asymptomatic infections and other factors. However, despite these limitations, the study presents important information on the disease burden and possible risk factors that may be involved with asymptomatic infections, which could be very useful for the formulation of effective strategies for surveillance, prevention and control of malaria transmission and be able to move towards its elimination.

Conclusion

The RT-MqPCR presented a high sensitivity and specificity in the detection of asymptomatic infections by *P. vivax* in comparison with the LM, which proved to be an insufficient technique for the active detection of patients with low parasitemia. Through this study, evidence is provided for the first time of the existence of a significant prevalence of asymptomatic infections by *P. vivax* in one of the main endemic regions of Panama. This clearly represents a new paradigm for the NMP that requires immediate attention, in order to maintain the achievements obtained in the fight against malaria to date and achieve the new objective of eliminating the disease in the country. RT-MqPCR, in addition to detecting asymptomatic infections, has the potential use of evaluating malaria vaccine candidates, detecting infections in blood donors, especially in endemic areas, epidemiological studies in endemic areas, monitoring and surveillance of autochthonous outbreaks in regions non-endemic, evaluate and monitor antimalarial treatments.

List of abbreviations NMP: National Malaria Program, **MINSA**: Ministerio de Salud, **ICGES**: Instituto Conmemorativo Gorgas de Estudios de la Salud, DE: Epidemiology Department, **LCRSP**: Central Public Health Reference Laboratory, **WHO**: World Health Organization, **PAHO:** Pan American Health Organization, **LM**: Light microscopy, **RDTs**: Rapid diagnostic tests, **PCR**: Polymerase chain reaction, **DNA**: Deoxyribonucleic acid, **CGY**: Comarca Guna Yala, **msnm**: meters above sea level

Ethics approval and consent to participate: The protocol was previously reviewed and approved by the MINSA NMP Institutional Technical Committee (Note No. 374/DCV/ICG/2016) and the Guna Yala Health Region (Note No. 25/DCV/DGS/MINSA/2016). The consent and approval of the traditional indigenous authorities (Sahila) of each of the selected communities was obtained through conversation, detailing the purpose, benefits and procedures of the work, everything was explained to each of the Sahilas in the local language (Guna) through a vector control technical staff belonging to this indigenous ethnic group. The taking of the thick blood film and filter paper samples was carried out as part of the routine work carried out by the vector control technical personnel of the Guna Yala Health Region, previously, as is customary, the consent of each volunteer was obtained after having received detailed information on the objectives of the work. Consent for minors under 18 years of age was obtained from the parent or guardian.

Consent for publication: Not applicable. Availability of data and materials. The data sets analyzed during the current study are available from the corresponding author on reasonable request.

Availability of data and materials: The data sets analyzed during the current study are available from the corresponding author on reasonable request.

Competing interests: The authors declare that they have no competing interests. LCC, AC and JC are members of the Sistema Nacional de Investigación (SNI), SENACYT, Panama. JLR Any opinions, findings, conclusions or recommendations expressed in this publication are those of the author(s) and do not necessarily reflect the view of the U.S. Department of Agriculture. The mention of firm names or trade products does not imply that they are endorsed or recommended by the U.S. Department of Agriculture over other firms or similar products not mentioned; the USDA is an equal opportunity employer.

Funding: This research was funded by the Ministry of Economy and Finance of Panama.

Authors' contributions: LCC conceived and designed the project, funding acquisition and project management, participated in field work as well as in the development of this study, analyzed data and interpreted the results, drafted the first version of the manuscript and final revision of the manuscript. AMS participated in laboratory work.

LCC and RT-C participated in camp work; LCC, RT-C, AMS and JLR analyzed data and interpreted the results. All the authors read, contributed and approved the final manuscript.

Acknowledgements: We would like to thank Jose Lasso (RIP), Chief of the Department of Vector Control from the Ministry of Health; Carlos Victoria (RIP), Director of the National Malaria Program; Luis De Urriola Director of CGY Health Region, Maibel Pérez Medical Director of the Playón Chico Health Center, Cipriano Ayerza regional coordinators of vector control; vector control technicians: Manuel Herrera, Eliverio López, Humberto Pérez, Arniel López, Zacarias Nelson and, Lester Porras. To the traditional indigenous authorities: Valerio Grimaldo the Ukupa, Lizandro López the Irgandi and Rolando Jimenez the Aidirgandi (RIP). Alberto Cumbrera for the development of maps and all the operative personnel that collaborated and supported the development of this research.

Authors information:

Lorenzo Caceres Carrera PhD. Departamento de Entomología Médica, Instituto Conmemorativo Gorgas de Estudios de la Salud, Panama City, Panama. Email: lcaceres@gorgas.gob.pa

Ana María Santamaría MSc. Departamento de Parasitología del Instituto Conmemorativo Gorgas de Estudios de la Salud. Panama City, Panama. Email: asantamaria@gorgas.gob.pa

Anakena Castillo PhD. Departamento de Entomología Médica, Instituto Conmemorativo Gorgas de Estudios de la Salud, Panama City, Panama. Email: acastillo@gorgas.gob.pa

Luis Romero Technical Microscopy. Laboratorio Central de Referencia de Salud Pública. Instituto Conmemorativo Gorgas de Estudios de la Salud. Ciudad de Panamá, Panamá. Email: lu20_49@yahoo.com

Eduardo Urriola Bachelor. Departamento de Entomología Médica, Instituto Conmemorativo Gorgas de Estudios de la Salud, Panama City, Panama. Email: eurriola@gorgas.gob.pa

José E. Calzad PhD. Departamento de Parasitología del Instituto Conmemorativo Gorgas de Estudios de la Salud. Panama City, Panama. Email: jcalzada@gorgas.gob.pa

Rolando Torres-Cosme Bachelor. Department of Medical Entomology, Gorgas Memorial Institute of Health Studies, Panama City, Panama. Email: rtorres@gorgas.gob.pa

References

1. **Hay SI, Guerra CA, Tatem AJ, Noor AM, Snow RW**. The global distribution and population at risk of malaria: past, present, and future. Lancet Infect Dis. 2004;4(6):327-36. doi: 10.1016/S1473-3099(04)01043-6.
2. **Al-Harti SA**. Assessment of three blood genomic-DNA preparation methods for malaria molecular diagnosis. J Egypt Soc Parasitol. 2016;46(1):1-8. doi: 10.12816/0026144. PMID: 27363035.
3. **Lindblade KA, Steinhardt L, Samuels A, Kachur SP, Slutsker L**. The silent threat: asymptomatic parasitemia and malaria transmission. Expert Rev Anti Infect Ther. 2013;11:623-39. doi: 10.1586/eri.13.45.
4. **Chen I, Clarke SE, Gosling R, Hamainza B, Killeen G, Magill A, et al**. Asymptomatic malaria: a chronic and debilitating infection that should be treated. PLoS Med. 2016;13:e1001942. doi: 10.1371/journal.pmed.1001942.
5. **Peto TJ, Tripura R, Lee SJ, Althaus T, Dunachie S, Nguon C, et al**. Association between subclinical malaria infection and inflammatory host response in a pre-elimination setting. PLoS ONE. 2016;11:e0158656. doi: 10.1371/journal.pone.0158656.
6. **World Health Organization**. Terminology of malaria and of malaria eradication. Report of a drafting committee. Geneva: World Health Organization; 1963. [Cited 2022 September 11]. Available from: https://apps.who.int/iris/handle/10665/39007.
7. **Rieckmann KH.** Asymptomatic malaria. Lancet 1970;1:82-3. doi: 10.1016/s0140-6736(70)91866-0.

8. **Bell D, Fleurent AE, Hegg MC, Boomgard JD, McConnico CC**. Development of new malaria diagnostics: matching performance and need. Malar J. 2016;15:406. doi.org/10.1186/s12936-016-1454-8.

9. **Harris I, Sharrock WW, Bain LM, Gray KA, Bobogare A, Boaz L, et al**. A large proportion of asymptomatic *Plasmodium* infections with low and sub-microscopic parasite densities in the low transmission setting of Temotu Province, Solomon Islands: challenges for malaria diagnostics in an elimination setting. Malar J. 2010;7;9:254. doi: 10.1186/1475-2875-9-254.

10. **Laishram DD, Sutton PL, Nanda N, Sharma VL, Sobti RC, Carlton JM, et al.** The complexities of malaria disease manifestations with a focus on asymptomatic malaria. Malar J. 2012;11:29. 2875-11-29. doi.org/10.1186/1475-2875-11-29.

11. **Sturrock HJ, Hsiang MS, Cohen JM, Smith DL, Greenhouse B, Bousema T, et al**. Targeting asymptomatic malaria infections: active surveillance in control and elimination. PLoS Med. 2013;10(6):e1001467. doi: 10.1371/journal.pmed.1001467.

12. **Okell LC, Bousema T, Griffin JT, Ouédraogo AL, Ghani AC, Drakeley CJ**. Factors determining the occurrence of submicroscopic malaria infections and their relevance for control. Nat Commun. 2012;3:1237. doi: 10.1038/ncomms2241.

13. **World Health Organization**. Guidelines for the treatment of malaria. 3rd ed. Geneva: World Health Organization; 2015. [Cited 2022 September 3]. Available from: https://apps.who.int/iris/bitstream/handle/10665/162441/9789241549127_eng.pdf;jsessionid=0D066A279183E02F10F1ACDBD34C7F44?sequence=1.

14. **Landier J, Haohankhunnatham W, Das S, Konghahong K, Christensen P, Raksuansak J, et al.** Operational Performance of a *Plasmodium falciparum*

Ultrasensitive Rapid Diagnostic Test for Detection of Asymptomatic Infections in Eastern Myanmar. J Clin Microbiol. 2018;26;56(8):e00565-18. doi: 10.1128/JCM.00565-18.

15. **Parker DM, Landier J, Thu AM, Lwin KM, Delmas G, Nosten FH; Malaria Elimination Task Force Group.** Scale up of a *Plasmodium falciparum* elimination program and surveillance system in Kayin State, Myanmar. Wellcome Open Res. 2017;22;2:98. doi: 10.12688/wellcomeopenres.12741.2.
16. **Sumari D, Mugasa J, Selemani M, Shekalaghe S, Mugittu K, Gwakisa P.** Prevalence of submicroscopic *Plasmodium falciparum* infections in asymptomatic children in low transmission settings in Bagamoyo, Tanzania. MWJ. 2016;7.
17. **Zimmerman PA, Howes RE**. Malaria diagnosis for malaria elimination. Curr Opin Infect Dis. 2015;28(5):446-54. doi: 10.1097/QCO.0000000000000191.
18. **Khairnar K, Martin D, Lau R, Ralevski F, Pillai DR**. Multiplex real-time quantitative PCR, microscopy and rapid diagnostic immuno-chromatographic tests for the detection of *Plasmodium spp*: performance, limit of detection analysis and quality assurance. Malar J. 2009;9;8:284. doi: 10.1186/1475-2875-8-284.
19. **Farcas GA, Zhong KJ, Mazzulli T, Kain KC**. Evaluation of the Real Art Malaria LC real-time PCR assay for malaria diagnosis. J Clin Microbiol 2004;42:636-8. doi: 10.1128/JCM.42.2.636-638.2004.
20. **Keen J, Farcas GA, Zhong K, Yohanna S, Dunne MW, Kain KC.** Real-time PCR assay for rapid detection and analysis of PfCRT haplotypes of chloroquine-resistant *Plasmodium falciparum* isolates from India. J Clin Microbiol 2007;45:2889-93. doi: 10.1128/JCM.02291-06.

21. **Ministerio de Salud de Panamá.** Boletín epidemiológico semanal de malaria 2022. [Cited 2023 April 25]. Available from: https://www.minsa.gob.pa/informacion-salud/malaria-2022.

22. **Guzmán HM, Guevara C, Castillo A**. Natural disturbances and mining of Panamanian coral reefs by indigenous people. Conserv Biol. 2003;17:1396-401.

23. **Empresa de Transmisión Eléctrica S.A**. Departamento de Hidrometeorología. Estadística panameña. 2017. [Cited 2022 June 15]. Available from: http://www.hidromet.com.pa/open_data.php?id=154020&t_est=M&clase_dato = 2&periodicidad=3&sensor=TEMP.

24. **Ministerio de Salud de Panamá**. Situación de la salud en Panamá. Documento marco. Panamá: Ministerio de Salud de Panamá; 2005. [Cited 2022 December 15]. Available from: http://www.minsa.gob.pa/sites/default/files/publicaciones/situacion_de_salud_panama_2013_0.pdf.

25. **Ministerio de Salud de Panamá.** Manual de normas y procedimientos para la malaria. Panamá: Ministerio de Salud de Panamá. 2011. [Cited 2022 April 15]. Available from: http://www.minsa.gob.pa/sites/default/files/.

26. **López-Antuñano FJ y G Schmunis**. Diagnóstico de Malaria. Organización Panamericana de la Salud. Publicación Científica No. 512. Washington: OPS-OMS; 1988; p. 39-50.

27. **Schellenberg JR, Smith T, Alonso PL, Hayes RJ.** What is clinical malaria? Finding case definitions for field research in highly endemic areas. Parasitol Today. 1994;10:439-42. doi.org/10.1016/0169-4758(94)90179-1

28. **Peters DH, Gray RH**. When is fever malaria? Lancet. 1992; 339:690.

29. **Bruce-Chwatt LJ**. Parasite index in malaria. Trans R Soc Trop Med Hyg 1958;52:389. doi.org/10.1016/0035-9203(58)90054-3.

30. **Calzada JE, Marquez R, Rigg C, Victoria C, De La Cruz M, Chaves LF, Cáceres L.** Characterization of a recent malaria outbreak in the autonomous indigenous region of Guna Yala, Panama. Malar J. 2015;17;14:459. doi: 10.1186/s12936-015-0987-6.

31. **Ferreira MU, Karunaweera ND, da Silva-Nunes M, da Silva NS, Wirth DF, Hartl, DL, et al**. Population structure and transmission dynamics of *Plasmodium vivax* in rural Amazonia. J. Infect. Dis. 2007;195,1218-26. doi: 10.1086/512685.

32. **Recht J, Siqueira AM, Monteiro WM, Herrera SM, Herrera S, Lacerda MVG**. Malaria in Brazil, Colombia, Peru and Venezuela: current challenges in malaria control and elimination. Malar J. 2017;4;16(1):273. doi: 10.1186/s12936-017-1925-6.

33. **Ganguly S, Saha P, Guha SK, Biswas A, Das S, Kundu PK, et al**. High prevalence of asymptomatic malaria in a tribal population in eastern India. J Clin Microbiol. 2013;51:1439-44. doi: 10.1128/JCM.03437-12.

34. **Lin JT, Saunders DL, Meshnick SR**. The role of submicroscopic parasitemia in malaria transmission: what is the evidence? Trends Parasitol. 2014;30(4):183-90. doi: 10.1016/j.pt.2014.02.004.

35. **Ladeia-Andrade S, Ferreira MU, de Carvalho ME, Curado I, Coura JR.** Age-dependent acquisition of protective immunity to malaria in riverine populations of the Amazon Basin of Brazil. Am J Trop Med Hyg 2009;80:452-9. doi: 10.4269/ajtmh.2009.80.452

36. **Alves FP, Gil LH, Marrelli MT, Ribolla PE, Camargo EP, Pereira-da-Silva LH.** Asymptomatic carriers of *Plasmodium spp*. as infection source for malaria vector mosquitoes in the Brazilian Amazon. J Med Entomol 2005;42(5):777-9. doi: 10.1093/jmedent/42.5.777.

37. **Beshir KB, Hallett RL, Eziefula AC, Bailey R, Watson J, Wright SG, et al.** Measuring the efficacy of anti-malarial drugs in vivo: quantitative PCR measurement of parasite clearance. Malar J. 2010;9,312. doi.org/10.1186/1475-2875-9-312.

38. **Bousema T, Okell L, Felger I, Drakeley C.** Asymptomatic malaria infections: detectability, transmissibility and public health relevance. Nat Rev Microbiol 2014;12:833–40. doi: 10.1038/nrmicro3364.

39. **Dokunmu TM, Adjekukor CU, Yakubu OF, Bello AO, Adekoya JO, Akinola O, Amoo EO, Adebayo AH**. Asymptomatic malaria infections and Pfmdr1 mutations in an endemic area of Nigeria. Malar J. 2019 Jun 27;18(1):218. doi: 10.1186/s12936-019-2833-8.

40. **Rajakaruna RS, Alifrangis M, Amerasinghe PH, Konradsen F**. Pre-elimination stage of malaria in Sri Lanka: assessing the level of hidden parasites in the population. Malar J. 2010;9,25. doi.org/10.1186/1475-2875-9-25.

41. **Fernando SD, Abeyasinghe RR, Galappaththy GN, Rajapaksa LC**. Absence of asymptomatic malaria infections in previously high endemic areas of Sri Lanka. Am J Trop Med Hyg. 2009;81:763-7. doi: 10.4269/ajtmh.2009.09-0042.

42. **Zoghi S, Mehrizi AA, Raeisi A, Haghdoost AA, Turki H, Safari R, et al**. Survey for asymptomatic malaria cases in low transmission settings of Iran under elimination programme. Malar J. 2012;11:126. doi: 10.1186/1475-2875-11-126.

43. **Atkinson JA, Johnson LM, Wijesinghe R, Bobogare A, Losi L, O'Sullivan M, et al**. Operational research to inform a subnational surveillance intervention for malaria elimination in Solomon Islands. Malar J. 2012;11:101. doi: 10.1186/1475-2875-11-101.
44. **Barrios ED, Russomando G, Puerto FD.** Absence of asymptomatic cases of malaria in a historically endemic indigenous locality of the Department of Caaguazu, Paraguay: moving toward elimination. Rev Soc Bras Med Trop. 2016;49:637-40. doi.org/10.1590/0037-8682-0197-2016
45. **Delgado-Ratto C, Soto-Calle VE, Van den Eede P, Gamboa D, Rosas A, Abatih EN, et al**. Population structure and spatio-temporal transmission dynamics of *Plasmodium vivax* after radical cure treatment in a rural village of the Peruvian Amazon. Malar J. 2014;13:8. doi.org/10.1186/1475-2875-13-8.
46. **Golassa L, Baliraine FN, Enweji N, Erko B, Swedberg G, Aseffa A.** Microscopic and molecular evidence of the presence of asymptomatic *Plasmodium falciparum* and *Plasmodium vivax* infections in an area with low, seasonal and unstable malaria transmission in Ethiopia. BMC Infect Dis. 2015;5;15:310. doi: 10.1186/s12879-015-1070-1.
47. **IA MG.** Epidemiology, malaria and pregnancy. Am J Trop Med Hyg. 1984;33:517–25. doi: 10.4269/ajtmh.1984.33.517.
48. **Ferreira UM & Castro CM.** Challenges for malaria elimination in Brazil. Malar J. 2016;15:284. doi: 10.1186/s12936-016-1335-1.

49. **Zhou G, Lo E, Zhong D, Wang X, Wang Y, Malla S, et al**. Impact of interventions on malaria in internally displaced persons along the China–Myanmar border: 2011–2014. Malar J. 2016;15;15:471. doi: 10.1186/s12936-016-1512-2.

50. **Li N, Parker DM, Yang Z, Fan Q, Zhou G, Ai G, et al.** Risk factors associated with slide positivity among febrile patients in a confict zone of northeastern Myanmar along the China–Myanmar border. Malar J. 2013;10;12:361. doi: 10.1186/1475-2875-12-361.

51. **Chourasia MK, Raghavendra K, Bhatt RM, Swain DK, Valecha N, Kleinschmidt I**. Burden of asymptomatic malaria among a tribal population in a forested village of central India: a hidden challenge for malaria control in India. Public Health. 2017;147:92-7. doi: 10.1016/j.puhe.2017.02.010.

52. **Walldorf JA, Cohee LM, Coalson JE, Bauleni A, Nkanaunena K, KapitoTembo A, et al.** School-age children are a reservoir of malaria infection in Malawi. PLoS ONE. 2015;10(7):e0134061. doi: 10.1371/journal.pone.0134061

53. **Schoeps A, Gabrysch S, Niamba L, Sie A, Becher H**. The efect of distance to health-care facilities on childhood mortality in rural Burkina Faso. Am J Epidemiol. 2011;173:492-8. doi: 10.1093/aje/kwq386.

54. **Pigeault R, Vezilier J, Cornet S, Zele F, Nicot A, Perret P, et al.** Avian malaria: a new lease of life for an old experimental model to study the evolutionary ecology of *Plasmodium.* Phil. Trans. R. Soc. B. 2015;370:20140300. doi.org/10.1098/rstb.2014.0300

55. **Sattabongkot J, Suansomjit C, Nguitragool W, Sirichaisinthop J, Warit S, Tiensuwan M, et al.** Prevalence of asymptomatic *Plasmodium* infections with sub-

microscopic parasite densities in the northwestern border of Thailand: a potential threat to malaria elimination. Malar J. 2018;12;17(1):329. doi: 10.1186/s12936-018-2476-1

56. **Bin Mohanna MA, Bin Ghouth AS, Rajaa YA**. Malaria signs and infection rate among asymptomatic schoolchildren in Hajr Valley, Yemen. East Mediterr Health J Egypt 2007;13(1):35-40. [Cited 2022 June 15]. Available from: https://apps.who.int/iris/handle/10665/117221.

57. **Clarke SE, Jukes MC, Njagi JK, Khasakhala L, Cundill B, Otido J, et al.** Effect of intermittent preventive treatment of malaria on health and education in schoolchildren: a cluster randomized, double-blind, placebo-controlled trial. Lancet 2008;12;372(9633):127-38. doi: 10.1016/S0140-6736(08)61034-X

58. **Njama-Meya D, Kamya MR, Dorsey G**. Asymptomatic parasitaemia as a risk factor for symptomatic malaria in a cohort of Ugandan children. Trop Med Int Health 2004;9(8):862-8. doi: 10.1111/j.1365-3156.2004.01277.x.

59. **Le Port A, Cot M, Etard J-F, Gaye O, Migot-Nabias F, Garcia A**. Relation between *Plasmodium falciparum* asymptomatic infection and malaria attacks in a cohort of Senegalese children. Malar J. 2008;7:193. doi: 10.1186/1475-2875-7-193.

60. **Falade CO, Ajayi IO, Nsungwa-Sabiiti J, Siribié M, Diarra A, Sermé L, et al**. Malaria rapid diagnostic tests and malaria microscopy for guiding malaria treatment of uncomplicated fevers in Nigeria and pre-referral cases in 3 African Countries. Clin Infect Dis. 2016;63(suppl 5):S290-7. doi: 10.1093/cid/ciw628.

61. **Pan American Health Organization**. Situación de la Malaria en la Región de las Américas, 2000–2012. Washington (USA): PAHO. 2013. [Cited 2023 January 23].

Available from: https://www.paho.org/hq/dmdocuments/2012/2012-cha-situacion-malaria-americas.pdf.

62. **Okell LC, Ghani AC, Lyons E, Drakeley CJ**. Submicroscopic infection in *Plasmodium falciparum*-endemic populations: a systematic review and meta-analysis. J Infect Dis. 2009;200:1509–17. doi: 10.1086/644781.

63. **Mfuh KO, Achonduh-Atijegbe OA, Bekindaka ON, Esemu LF, Mbakop CD, Gandhi K, et al.** A comparison of thick-film microscopy, rapid diagnostic test, and polymerase chain reaction for accurate diagnosis of *Plasmodium falciparum* malaria. Malar J. 2019;12;18(1):73. doi: 10.1186/s12936-019-2711-4.

64. **World Health Organization**. Malaria microscopy quality assurance manual – versión 2. Geneva, Switzerland. Geneva. 2016. [Cited 2022 July 23]. Available from: https://www.who.int/malaria/publications/atoz/9789241549394/en/.

65. **Tada MS, Marques RP, Mesquita E, Martha RCD, Rodrigues JA, Costa JDAN, et al.** Urban malaria in the Brazilian Western Amazon Region I. High prevalence of asymptomatic carriers in an urban riverside district is associated with a high level of clinical malaria. Mem Inst Oswaldo Cruz. 2007;102:263–9. doi: 10.1590/s0074-02762007005000012.

66. **Vitor-Silva S, Siqueira AM, de Souza SV, Guinovart C, Reyes-Lecca RC, de Melo GC, et al.** Declining malaria transmission in rural Amazon: changing epidemiology and challenges to achieve elimination. Malar J. 2016;15:266. doi: 10.1186/s12936-016-1326-2.

67. **Barbosa S, Gozze AB, Lima NF, Batista CL, Bastos MDS, Nicolete VC, et al**. Epidemiology of disappearing *Plasmodium vivax* malaria: a case study in rural Amazonia. PLoS Negl Trop Dis. 2014;8:e3109. doi: 10.1371/journal.pntd.0003109.

68. **Kuamsab N, Putaporntip C, Pattanawong U, Jongwutiwes S**. Simultaneous detection of Plasmodium vivax and *Plasmodium falciparum* gametocytes in clinical isolates by multiplex-nested RT-PCR. Malar J. 2012;10;11:190. doi: 10.1186/1475-2875-11-190.

69. **Lima NF, Bastos MS, Ferreira MU**. *Plasmodium vivax*: reverse transcriptase real-time PCR for gametocyte detection and quantitation in clinical samples. Exp Parasitol. 2012;132:348–54. doi: 10.1016/j.exppara.2012.08.010.

70. **Fançony C, Sebastião YV, Pires JE, Gamboa D, Nery SV**. Performance of microscopy and RDTs in the context of a malaria prevalence survey in Angola: a comparison using PCR as the gold standard. Malar J. 2013;12:284. doi.org/10.1186/1475-2875-12-284

71. **Wongsrichanalai C, Barcus MJ, Muth S, Sutamihardja A, Wernsdorfer WH**. A review of malaria diagnostic tools: microscopy and rapid diagnostic test (RDT). Am J Trop Med Hyg. 2007;77(6 Suppl):119–27.

72. **Berzosa P, de Lucio A, Romay-Barja M, Herrador Z, González V, García L, et al.** Comparison of three diagnostic methods (microscopy, RDT, and PCR) for the detection of malaria parasites in representative samples from Equatorial Guinea. Malar J [Internet]. 2018;77;17(1):333. [Cited 2022 September 3]. Available from: https://pubmed.ncbi.nlm.nih.gov/30223852.

73. **Sumari D, Mwingira F, Selemani M, Mugasa J, Mugittu K, and Gwakisa P**. Malaria prevalence in asymptomatic and symptomatic children in Kiwangwa, Bagamoyo district, Tanzania. Malar J. 2017;25;16(1):222. doi: 10.1186/s12936-017-1870-4.

74. **Zaw MT, Thant M, Hlaing TM, Aung NZ, Thu M, Phumchuea K, et al**. Asymptomatic and sub-microscopic malaria infection in Kayah State, eastern Myanmar. Malar J. 2017;4;16(1):138. doi: 10.1186/s12936-017-1789-9.

75. **Chourasia MK, Raghavendra K, Bhatt RM, Swain DK, Valecha N, Kleinschmidt I.** Burden of asymptomatic malaria among a tribal population in a forested village of central India: a hidden challenge for malaria control in India. Public Health. 2017;147:92–7. doi: 10.1016/j.puhe.2017.02.010.

76. **Zhao Y, Zhao Y, Lv Y, Liu F, Wang Q, Li P, et al**. Comparison of methods for detecting asymptomatic malaria infections in the China-Myanmar border area. Malar J. 2017;20;16(1):159. doi: 10.1186/s12936-017-1813-0.

77. **Shute GT, Sodeman TM**. Identification of malaria parasites by fluorescence microscopy and acridine orange staining. Bull World Health Organ 1973; 8: 591-96.

78. **Johnston SP, Pieniazek NJ, Xayavong M V., Slemenda SB, Wilkins PP, Da Silva AJ**. PCR as a confirmatory technique for laboratory diagnosis of malaria. J Clin Microbiol. 2006;44(3):1087 9.

79. **Rantala AM, Taylor SM, Trottman PA, Luntamo M, Mbewe B, Maleta K, et al.** Comparison of real-time PCR and microscopy for malaria parasite detection in Malawian pregnant women. Malar J. 2010;6;9:269. doi: 10.1186/1475-2875-9-269.

80. **Andrade ALSS, Martelli CMT, Oliveira RM, Arias JR, Zicker F, Pang L.** High prevalence of asymptomatic malaria in Gold Mining areas in Brazil. Clin Infec Dis 1995;20: 475. doi: 10.1093/clinids/20.2.475.

81. **Suárez-Mutis MC, Bonilla MP, Blandón ME**. Prevención y control de la malaria entre los Yujup-Maku, de la Libertad: un grupo étnico al borde de la extinción. Resúmenes 2 XV Congreso Internacional de Medicina Tropical e Higiene y Malaria, Cartagena. 2000b. p. 113.

82. **Suárez-Mutis MC, Cuervo P, FMS L, Moraes-Avila SL, Ferreira AW, Fernandes O, et al.** Cross sectional study reveals a high percentage of asymptomatic *Plasmodium vivax* infection in the Amazon Rio Negro area, Brazil. Rev Inst Med Trop. 2007;49:159-64. doi: 10.1590/s0036-46652007000300005.

83. **Roshanravan B, Ari E, Gilman RH, Cabrera L, Lee E, Metclafe J, et al.** Endemic malaria in the Peruvian Amazon region of Iquitos. Am J Trop Med Hyg. 2003;69:45-52.

84. **Suárez-Mutis MC, Cuervo P, Fernandes O, Coura JR**. Evidência da presença de infecção assintomática por *Plasmodium* no médio rio Negro, Estado do amazonas. Rev Soc Bras Med Trop 37 (Supl. I). 2004;268-9.

85. **Wide A, Pabón R, De Abreu N, Bargues MD, Salcedo A, Capaldo J, et al.** Prevalence of asymptomatic *Plasmodium vivax* infections in the north-eastern focus of malaria of Venezuela. Bol Mal Salud Amb. 2016;56(2):160-71.

86. **Vásquez-Jiménez JM, Arévalo-Herrera M, Henao-Giraldo J, Molina-Gómez K, Arce-Plata M, Vallejo AF, et al**. Consistent prevalence of asymptomatic infections

in malaria endemic populations in Colombia over time. Malar J. 2016;6;15:70. doi: 10.1186/s12936-016-1124-x.

87. **Rovira-Vallbona E, Contreras-Mancilla JJ, Ramirez R, Guzmán-Guzmán M, Carrasco-Escobar G, Llanos-Cuentas A, et al**. Predominance of asymptomatic and sub-microscopic infections characterizes the *Plasmodium* gametocyte reservoir in the Peruvian Amazon. PLoS Negl Trop Dis. 2017;3;11(7):e0005674. doi: 10.1371/journal.pntd.0005674.

88. **Yin JH, Yan H, Huang F, Li M, Xiao HH, Zhou SS, et al**. Establishing a China malaria diagnosis reference laboratory network for malaria elimination. Malar J. 2015;14:40. doi: 10.1186/s12936-015-0556-z.

89. **Poschl B, Waneesorn J, Thekisoe O, Chutipongvivate S, Karanis P**. Comparative diagnosis of malaria infections by microscopy, nested PCR, and LAMP in northern Thailand. Am J Trop Med Hyg. 2010;83:56–60. doi: 10.4269/ajtmh.2010.09-0630.

90. **Anthony C, Mahmud R, Lau YL, Syedomar SF, La Sri Sri, Ponnampalavanar S**. Comparison of two nested PCR methods for the detection of human malaria. Trop Biomed. 2013;30:459-66.

91. **Alemayehu S, Feghali KC, Cowden J, Komisar J, Ockenhouse CF, Kamau E**. Comparative evaluation of published real-time PCR assays for the detection of malaria following MIQE guidelines. Malar J. 2013;12:277. doi: 10.1186/1475-2875-12-277.

92. **Cheng Z, Wang D, Tian X, Sun Y, Sun X, Xiao N, et al**. Capture and ligation probe-PCR (CLIP-PCR) for molecular screening, with application to active malaria

surveillance for elimination. Clin Chem. 2015;61:821–8. doi: 10.1373/clinchem.2014.237115.

93. **Hopkins H, González IJ, Polley SD, Angutoko P, Ategeka J, et al.** Highly Sensitive Detection of Malaria Parasitemia in a Malaria-Endemic Setting: Performance of a New Loop-Mediated Isothermal Amplification Kit in a Remote Clinic in Uganda. J Infect Dis. 2013;15;208(4):645-52. doi: 10.1093/infdis/jit184.

94. **Rojas-Jaimes J.** Detección de malaria usando reacción en cadena de la polimerasa en los distritos de Huepetuhe y Colorado, Madre de Dios. Rev. Peru. Epidemiol. 2014;18:1-5.

95. **Oaks SC, Mitchell VS, Pearson GW, Carpenter CCJ.** Malaria. Obstacles and opportunities. National Academy of Science 1991; Press, Washington, DC.

96. **Dajem SM**. Molecular investigation of mixed malaria infections in Southwest Saudi Arabia. Saudi Med J. 2015;36:248. doi.org/10.15537/smj.2015.2.10874.

97. **Gupta B, Gupta P, Sharma A, Singh V, Dash AP, Das A**. High proportion of mixed-species *Plasmodium* infections in India revealed by PCR diagnostic assay. Trop Med Int Health. 2010;15:819-24. doi.org/10.1111/j.1365-3156.2010.02549.x.

98. **Krishna S, Bharti PK, Chandel HS, Ahmad A, Kumar R, Singh PP, et al.** Detection of mixed infections with *Plasmodium spp*. by PCR, India, 2014. Emerg Infect Dis. 2015;21:1853. doi.org/10.3201/ eid2110.150678

99. **Mendoza NM, Montoya R, García M, Padilla JC, Bruzón LO, Mendoza E, et al.** Evaluación de campo de una prueba rápida para el diagnóstico de malaria. Biomédica. 2001;21:313-9. doi.org/10.7705/biomedica.v21i4.1123

100. **Montoya AE, Menco J, Osorio N, Zuluaga MA, Duque J, et al.** Concordancia entre gota gruesa, inmunocromatografía y reacción en cadena de la polimerasa para el diagnóstico de malaria. Biomédica 2008;28:252-61. doi.org/10.7705/biomedica.v28i2.96.

101. **Nafo F.** Rolling back malaria: Opportunities and challenges. Trans R Soc Trop Med Hyg. 2005;99:403-6. doi. org/10.1016/j.trstmh.2005.02.002

102. **Hetzel MW, Iteba N, Makemba A, Mshana C, Lengeler C, Obrist B, *et al*.** Understanding and improving access to prompt and effective malaria treatment and care in rural Tanzania: the ACCESS Programme. Malar J. 2007;6:83. ¿doi.org/10.1186/1475-2875-6-83

103. **Cáceres Carrera L, Victoria C, Ramirez JL, Jackman C, Calzada JE, Torres R**. Study of the epidemiological behavior of malaria in the Darien Region, Panama. 2015-2017. PLoS One. 2019 Nov 15;14(11):e0224508. doi: 10.1371/journal.pone.0224508.

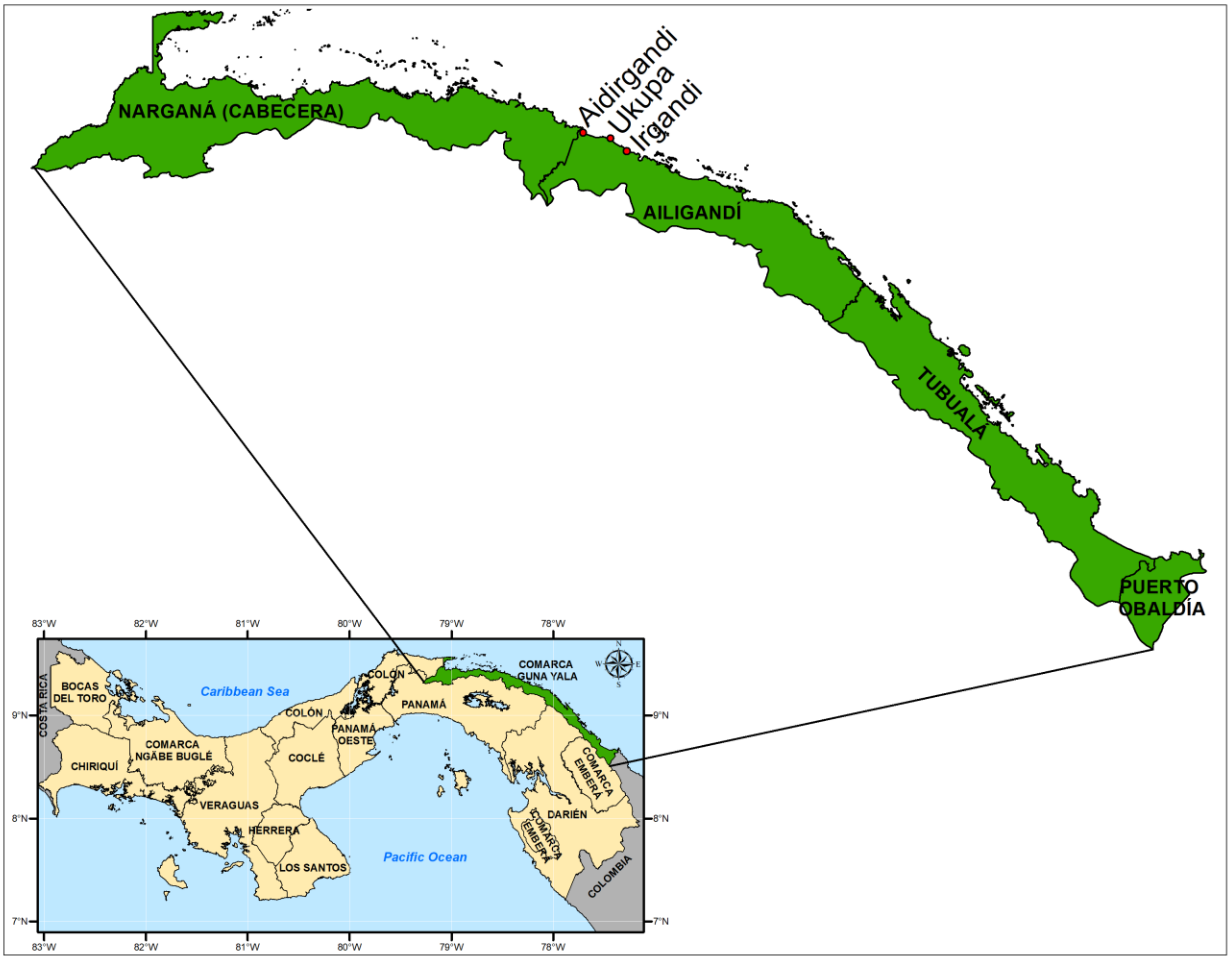

Fig 1. Map showing the geographic location of the communities of Irgandi, Ukupa and Aidirgandi in the Guna Yala Comarca, Panama

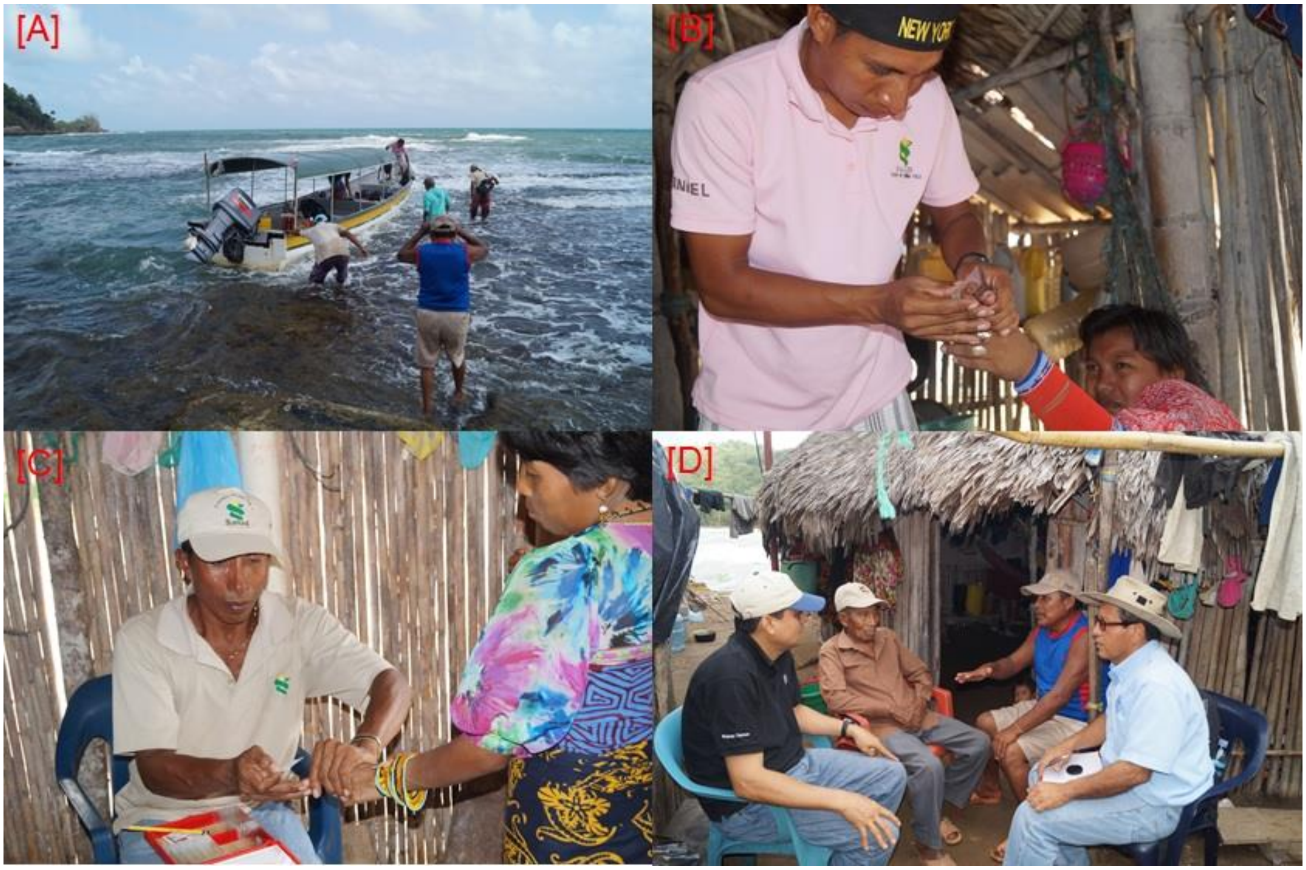

Fig 2. Research team arrived to Irgandi community [A], thick blood sample collection [B], filter paper blood sample collection [C] and Research team met with Lizandro López, sahila of the Irgandi community [D].

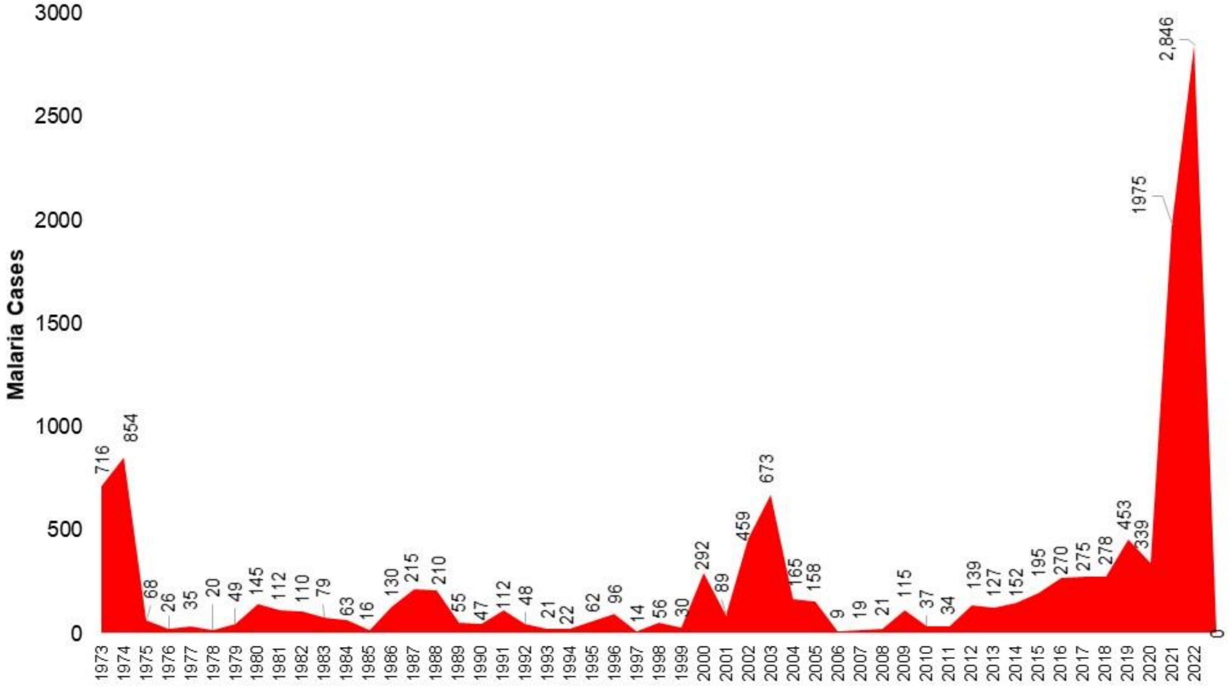

Fig 3. Epidemiological behavior of malaria in the Guna Yala Comarca, Panama. 1973 – 2022. Source MINSA, Panama.

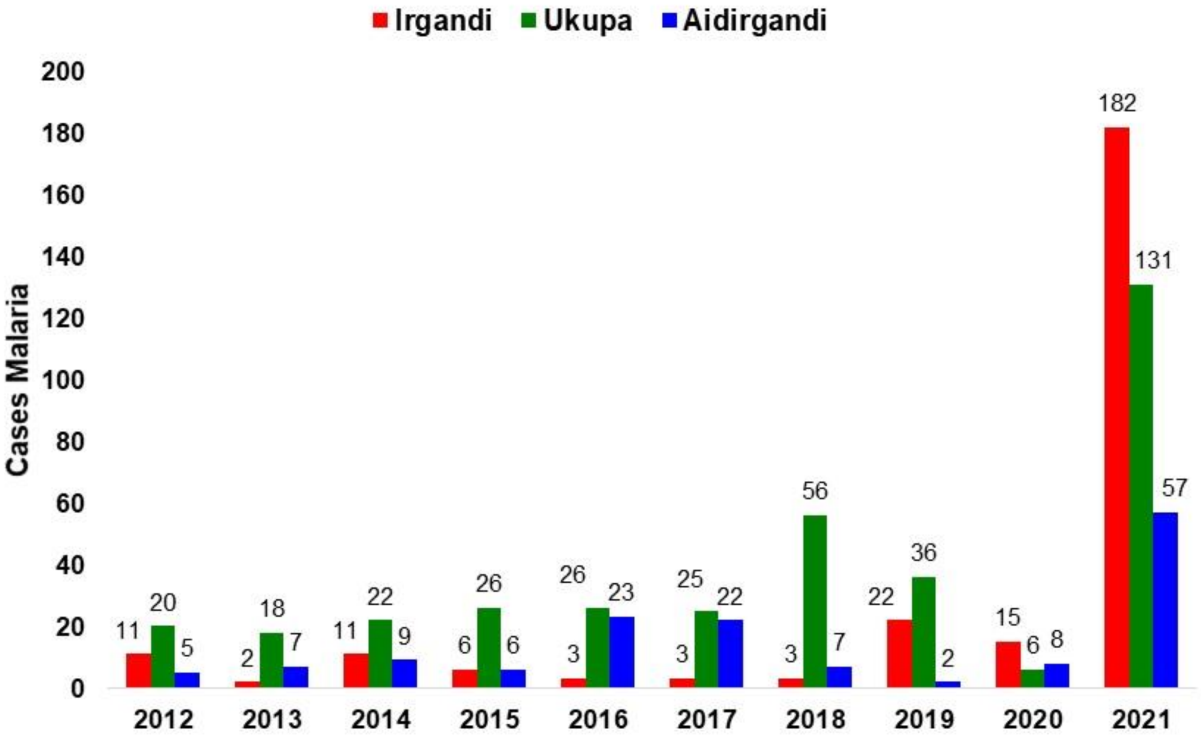

Fig 4. Malaria cases registered in the communities of Irgandi, Ukupa and Aidirgandi, Guna Yala Comarca, Panama. 2012 – 2021.

Table 1. Number of blood samples taken by age group and sex for the diagnosis of asymptomatic malaria by LM and RT-MqPCR in the three communities in the Guna Yala Comarca, Panama.

Age Group	Irgandi (N=300 habitants)					Ukupa (N=320 habitants)					Aidirgandi (N=190 habitants)					Total Samples	% Samples
	Male	Femen	Total Samples	% Samples	% Samples comm	Male	Femen	Total Samples	% Samples	% Samples comm	Male	Femen	Total Samples	% Samples	% Samples comm		
< 1 a 10	35	35	70	39.1		42	34	76	36.0		35	25	60	37.3		206	37.4
11 a 20	16	29	45	25.1		42	29	71	33.6		19	22	41	25.5		157	28.5
21 a 30	9	14	23	12.8		9	12	21	10.0		1	17	18	11.2		62	11.2
31 a 40	4	8	12	6.7	60%	3	9	12	5.7	66%	7	13	20	12.4	85%	44	8.0
41 a 50	2	6	8	4.5		7	10	17	8.1		2	6	8	5.0		33	6.0
51 a 60	3	7	10	5.6		3	5	8	3.8		1	10	11	6.8		29	5.3
61 a 70	3	5	8	4.5		5	1	6	2.8		0	0	0	0.0		14	2.5
> 71	1	2	3	1.7		0	0	0	0		1	2	3	1.8		6	1.1
Total	40.8% 73/179	59.2% 106/179	32.5% 179/551	100	179/300	52.6% 111/211	47.4% 100/211	38.3% 211/551	100	211/320	41.0% 66/161	59.0% 95/161	29.2% 161/551	100	161/190	551	100

cmm: Community

Table 2. Parasite density of asymptomatic infections diagnosed by LM in the three communities of the Guna Yala Comarca, Panama.

Patient	Microscopy	Gametocyte Number	RT-MqPCR	Parasitemia	Nivel de Parasitemia
1	*P. vivax*	30	P. vivax	3,571 parasitos/µl sangre	
2	*P. vivax*	25	P. vivax	3,571 parasitos/µl sangre	
3	*P. vivax*	8	P. vivax	2,631 parasitos/µl sangre	Moderada
4	*P. vivax*	20	P. vivax	1,692 parasitos/µl sangre	
5	*P. vivax*	3	P. vivax	1,541 parasitos/µl sangre	
6	*P. vivax*	0	P. vivax	301 parasitos/µl sangre	
7	*P. vivax*	0	P. vivax	113 parasitos/µl sangre	
8	*P. vivax*	0	P. vivax	94 parasitos/µl sangre	
9	*P. vivax*	0	P. vivax	56 parasitos/µl sangre	Baja
10	*P. vivax*	0	P. vivax	45 parasitos/µl sangre	
11	*P. vivax*	0	P. vivax	45 parasitos/µl sangre	
12	*P. vivax*	0	P. vivax	38 parasitos/µl sangre	
13	*P. vivax*	0	P. vivax	26 parasitos/µl sangre	

Table 3. Diagnosis by LM and RT-MqPCR of asymptomatic patients of *P. vivax* and its prevalence in the three communities in the Guna Yala Comarca, Panama.

Age Group	Irgandi				Ukupa								Aidirgandi							
	RT-MqPCR	Sex		% Prev	ML	Sex		% Prev	RT-MqPCR	Sex		% Prev		Sex		% Prev	RT-MqPCR	Sex		% Prev
		M	F			M	F			M	F		ML	M	F			M	F	
< 1 a 10	7	3	4		4	2	2		23	12	11		3	2	1		8	4	4	
11 a 20	1		1		2	1	1		12	7	5		2	1	1		4	3	1	
21 a 30	2	1	1		1	1			3	1	2						3		3	
31 a 40				3.3				2.2	3		3	13.4	1	1		3.2	3	2	1	12.6
41 a 50																				
51 a 60									1		1						1		1	
61 a 70									1	1										
> 71																	2	1	1	
**TOTAL**	**10**	**4**	**6**	**3.3**	**7**	**4**	**3**	**2.2**	**43**	**21**	**22**	**13.4**	**6**	**4**	**2**	**3.2**	**21**	**10**	**11**	**12.6**

LM: Light microscopy, % Prev: % Prevalence
