# Supplementary material for: Detection through the use of RT-MqPCR of asymptomatic reservoirs of malaria in samples of patients from the indigenous Comarca of Guna Yala, Panama: Essential method to achieve the elimination of malaria
Source: PLoS One. 2024 Jul 24;19(7):e0305558. doi: 10.1371/journal.pone.0305558 (PMC11268588; doi:10.1371/journal.pone.0305558)
Supplement: Attachment — Submitted filename: Response to Reviewers - 4.pdf [file pone.0305558.s002.pdf]

Panama, January 26, 2026

Doctor
Maidelyn R. Peregrin
Academic Editor
PLOS ONE

Dear Dr. Peregrin:

We hereby forward to you the corrections made to the manuscript [PONE-D-23-20941] - [EMID:91dfbf0d0b91cc2d] titled "Detection through the use of RT-MqPCR of asymptomatic reservoirs of malaria in samples of patients from the indigenous Comarca of Guna Yala, Panama: Essential method to achieve the elimination of malaria", with corrections made according to the observations of the Plos One editor and evaluators.

Editorial review:

1. The manuscript was corrected according to the style requirements of Plos One.
2. The manuscript was edited to improve language, spelling and grammar. Dr. José Luis Ramirez revised the language, spelling and grammar of the manuscript.
3. The financial information in the "Financing Information" and "Financial Disclosure" sections was corrected. This research work was funded by the Ministerio de Economía y Finanzas de Panama with Gram No. 9044-052.
4. The availability of information to be included in the "Repository" will be made when requested by Plos One.
5. The declaration of ethics was modified according to the recommendations and style of Plos One.
6. Fig 1 map was prepared by the Geographic Information Unit of Gorgas Memorial Institute for Health Studies. License by ESRI ArcGIS Online and ArcGIS Desktop 10.7.1 and ESRI Pro.
7. We indicate that there is no ethical or legal restriction to provide the data related to this study.
8. The data was uploaded to the figshare repository whit URL: https://figshare.com/articles/dataset/Detection_through_the_use_of_RT-MqPCR_of_asymptomatic_reservoirs_of_malaria_in_samples_of_patients_from_the_indigenous_Comarca_of_Guna_Yala_Panama_Essential_method_to_achieve_the_elimination_of_malaria/24885660
9. In the previously sent letter, it was indicated that Fig 2 was removed. Considering that only verbal consent was obtained from the photographed people and the PLOS consent form for publication in a PLOS journal has not been signed by each of the people.
10. The methodology was modified where photographs were mentioned, and ethical considerations were also modified.

Reviewer 1

Line 59: The indicated prevalence percentage is the average in the three communities (27.8/3, 9.3%), The value 13.4% is the prevalence observed in Ukupa community.
Line 60: We confirm that 3.3% is the prevalence for the Irgandi community.
Line 65: The age group was corrected: 1 to 21 years.
Line 101: The paragraph that begins on line 99 and ends on line 103 belongs to textually to the same reference.
Line 111: Added the requested reference.
Line 117: In the reference, 200 parasites/ml is textually indicated.
Line 128: The text "polymerase chain reaction (PCR)" was added.
Line 135: In the reference, parasites/ml is indicated textually.
Line 137: We added nested PCR (nPCR) text.
Line 163: We added the requested reference, starting from line 161 to line 163.
Line 194: The °C sign was added.
Línea 203: Parasite added.
Line 211: We added the requested reference.
Line 216: The methodology indicates the type of sampling used.
Line 223: Corresponds to the sign of degrees Celsius °C.
Line 251: The word "Minors" was changed to "children".
Line 259: Average 10 µl blood was added.
Line 281: RT-MqPCR was rewritten.
Line 282: DNA was extracted, the kit used and the amount of DNA that was extracted were added.
Line 282: RT-MqPCR target added.
Line 345: Added months to those less than a year (4 to 11 months)
Line 353: Added months to those less than a year (5 to 8 months)
Line 356: Added months to those less than a year (4 to 11 months)
Line 359: Added months to those less than a year (4 to 7 months)
Line 378: "µl" was added.
Line 383: Corrected age group to 1 to 12 years of age.
Line 386: Attach amplification curve graphs.
Line 394: The text and Table 3 were corrected, and reference is made to the inhabitants.
Line 457: The reference to the epidemiological study carried out in the CKY was placed.
Line 1215: Improved description of Fig 1 (map)
Line 1225: Improved the title of Fig 2.
Line 1237: Improved the title of Fig 3.
Line 1261: The description of children <1 year (4 to 11 months) was added in the bottom of Table 1.
Line 1277: In the discussion, reference is made to the distribution of cases.
Line 1279 Table 2 was renovated and corrected.

Reviewer 2.

1. The introduction was improved and paragraphs with repeated information were eliminated.
2. Statistical analysis was improved.
3. Table 2 was renovated and corrected.
4. The conclusion was corrected.
5. The summary was improved.
6. Line 233: Improved the operational definition of symptomatic and asymptomatic malaria.
7. The data in results was verified and corrected.
8. Line 351: IC was removed.
9. Table 2 title was improved.
10. The study was carried out in 2016 and reference is being made to the Irgandi cases in 2021.
11. Table 3 was renovated and improved.
12. Before taking blood samples from the participants, one of the questions asked was if they had migrated somewhere in the last two weeks, everyone's answer was no.
13. Line 525: In the revised manuscript this information was eliminated.

Thanking you for your attention, sincerely,

Dr. Lorenzo Cáceres Carrera
Senior health researcher of the ICGES
