# Supplementary material for: Detection through the use of RT-MqPCR of asymptomatic reservoirs of malaria in samples of patients from the indigenous Comarca of Guna Yala, Panama: Essential method to achieve the elimination of malaria
Source: PLoS One. 2024 Jul 24;19(7):e0305558. doi: 10.1371/journal.pone.0305558 (PMC11268588; doi:10.1371/journal.pone.0305558)
Supplement: Attachment — Submitted filename: Response to Reviewers.pdf [file pone.0305558.s003.pdf]

IGORGAS | INSTITUTO CONMEMORATIVO GORGAS DE ESTUDIOS DE LA SALUD (1928)

17 de AGOSTO 95 Aniversario 1928-2023

Panama, May 29, 2024

Doctor
Luzia H Carvalho
Academic Editor
PLOS ONE

Dear Doctor. Carvalho:

We hereby forward to you the corrections made to the manuscript [PONE-D-23-20941] - [EMID:91dfbf0d0b91cc2d] titled "Detection through the use of RT-MqPCR of asymptomatic reservoirs of malaria in samples of patients from the indigenous Comarca of Guna Yala, Panama: Essential method to achieve the elimination of malaria", with corrections made according to the observations of the Plos One editor and evaluators.

Editorial review:

1. The language was appropriately adjusted through Editage services, we attach a certificate of editing of the manuscript.
2. The list of references was reviewed and repeated ones replaced.

Reviewer 1

1. Table 1 - correct "Femen" to female
2. Table 2 - correct "porcentaje" to percentage

Thanking you for your attention, sincerely,

Dr. Lorenzo Cáceres Carrera
Senior health researcher at ICGES

(507) 527-4800 I igorgas@gorgas.gob.pa I Av. Justo Arosemena, entre calle 35 y 36, Cd. de Panamá
